# Effects of acute ischemic stroke on binaural perception

**DOI:** 10.3389/fnins.2022.1022354

**Published:** 2022-12-22

**Authors:** Anna Dietze, Peter Sörös, Matthias Bröer, Anna Methner, Henri Pöntynen, Benedikt Sundermann, Karsten Witt, Mathias Dietz

**Affiliations:** ^1^Department of Medical Physics and Acoustics, University of Oldenburg, Oldenburg, Germany; ^2^Cluster of Excellence “Hearing4all”, University of Oldenburg, Oldenburg, Germany; ^3^Department of Neurology, School of Medicine and Health Sciences, University of Oldenburg, Oldenburg, Germany; ^4^Research Center Neurosensory Science, University of Oldenburg, Oldenburg, Germany; ^5^Institute of Radiology and Neuroradiology, Evangelisches Krankenhaus Oldenburg, Oldenburg, Germany

**Keywords:** binaural hearing, psychoacoustics, brain lesions, lateralization, binaural masking level difference, magnetic resonance imaging, stroke

## Abstract

Stroke-induced lesions at different locations in the brain can affect various aspects of binaural hearing, including spatial perception. Previous studies found impairments in binaural hearing, especially in patients with temporal lobe tumors or lesions, but also resulting from lesions all along the auditory pathway from brainstem nuclei up to the auditory cortex. Currently, structural magnetic resonance imaging (MRI) is used in the clinical treatment routine of stroke patients. In combination with structural imaging, an analysis of binaural hearing enables a better understanding of hearing-related signaling pathways and of clinical disorders of binaural processing after a stroke. However, little data are currently available on binaural hearing in stroke patients, particularly for the acute phase of stroke. Here, we sought to address this gap in an exploratory study of patients in the acute phase of ischemic stroke. We conducted psychoacoustic measurements using two tasks of binaural hearing: binaural tone-in-noise detection, and lateralization of stimuli with interaural time- or level differences. The location of the stroke lesion was established by previously acquired MRI data. An additional general assessment included three-frequency audiometry, cognitive assessments, and depression screening. Fifty-five patients participated in the experiments, on average 5 days after their stroke onset. Patients whose lesions were in different locations were tested, including lesions in brainstem areas, basal ganglia, thalamus, temporal lobe, and other cortical and subcortical areas. Lateralization impairments were found in most patients with lesions within the auditory pathway. Lesioned areas at brainstem levels led to distortions of lateralization in both hemifields, thalamus lesions were correlated with a shift of the whole auditory space, whereas some cortical lesions predominantly affected the lateralization of stimuli contralateral to the lesion and resulted in more variable responses. Lateralization performance was also found to be affected by lesions of the right, but not the left, basal ganglia, as well as by lesions in non-auditory cortical areas. In general, altered lateralization was common in the stroke group. In contrast, deficits in tone-in-noise detection were relatively scarce in our sample of lesion patients, although a significant number of patients with multiple lesion sites were not able to complete the task.

## 1. Introduction

The interaural level differences (ILD) and interaural time differences (ITD) provide the basis for localizing sound sources in the horizontal plane. This ability informs the listener about the spatial location of an approaching vehicle, for instance, but is also crucial for segregating different auditory streams in more complex listening environments, such as multiple talkers in a crowded restaurant. Especially the latter ability is clearly compromised in listeners with sensorineural hearing loss (e.g., Gatehouse, 2004; Shinn-Cunningham and Best, 2008). However, spatial hearing can also be impaired by damage to the central nervous system. The consequences of such damage for spatial hearing and binaural perception are arguably less well understood (Gallun, 2021).

One relatively prevalent type of central nervous system damage is stroke. For instance, the GEDA 2014/2015-EHIS study found that, in Germany, 1.6% of adults suffered a stroke or chronic consequences of a stroke during the past 12 months (Robert Koch-Institut, 2017). Central stroke lesions do not usually affect hearing thresholds, but they can affect binaural hearing (Häusler and Levine, 2000). This is also reflected in patient-reported difficulties in sound localization in the chronic phase after stroke, as shown in Bamiou et al. (2012). Given the relatively high prevalence of stroke in the general population, an improved understanding of its effects on spatial hearing would be desirable.

Previous studies have revealed deficits in binaural hearing in patients with different stroke lesion locations. Furst et al. (2000) investigated the binaural performance of patients with brainstem lesions using a test of interaural difference discrimination and with a lateralization task. Binaural performance was affected whenever the lesion overlapped the auditory pathway. Lesions of the caudal pons led to center-oriented lateralization, whereas lesions rostral to the superior olivary complex led to side-oriented lateralization results. Just-noticeable differences in ILD and ITD were affected in some patients with pontine lesions.

Comparable methods were used by Spierer et al. (2009), who studied the effects of cortical lesions on ITD- and ILD-based lateralization. The findings suggested a dominance of the right hemisphere in auditory spatial representation. More frequent and more severe deficits were observed after right-sided, compared to left-sided, damage. Lesions of the right hemisphere influenced contralesional as well as ipsilesional lateralization, whereas the effect of left-sided damage was restricted mainly to the contralesional hemifield.

Along the same lines, the effect of auditory neglect (impaired perception of auditory stimuli in one hemispace) is also more frequently observed for right-hemispheric lesions, especially when the temporal lobe is damaged (Gokhale et al., 2013). The term neglect is used for various impairments and different modalities (Heilman et al., 2000). As reviewed in Gokhale et al. (2013), language-related stimuli are mainly associated with the left temporal cortex, whereas non-language stimuli are predominantly processed in the right hemisphere. As a result, processing of non-language stimuli is often impaired, and in some cases, neglected after damage to the right hemisphere.

Two separate processing streams are suspected to be responsible for the ‘where’ and ‘what’ of auditory perception. This hypothesis is supported by the fact that binaural hearing performance of the centrally impaired auditory system depends not only on the location of the damaged area, but also on the task to be performed (Bellmann et al., 2001). For instance, a case report of a patient with lesions in the right hemisphere showed a difference between using binaural cues implicitly or explicitly (Thiran and Clarke, 2003). The patient was able to implicitly use binaural cues for stream segregation in a spatial-release-from-masking task, but had no explicit lateralized perception at all when presented with stimuli with ITDs. The implicit and explicit use of binaural cues was also investigated by Tissieres et al. (2019), with a larger number of participants. They concluded that the implicit use of auditory spatial cues relies on a distinct, left-dominated network.

In general, previous studies on the effect of lesions of the central nervous system on binaural perception were mainly investigated in the chronic phase of stroke in subgroups of stroke populations. Based on the results of, e.g., Trapeau and Schönwiesner (2015), who showed that relearning of localization with altered ITDs is possible within a few days, we assume that stroke-induced lateralization impairments will be strongest in the acute phase and at least partially recovered in the chronic phase of stroke. The existing studies revealed a plethora of deficits that vary significantly across lesion location, stimulus material and patients. The great variability and individual nature of the findings indicate that further large-scale research is needed to move closer to a complete understanding of the effects of stroke on binaural hearing performance. By studying the disturbed system shortly after stroke onset, the patients’ responses may give novel insights into the role of the affected areas in spatial hearing, including its relevance for the healthy system.

In addition to studies with stroke patients, neuroscientific experiments with healthy adults revealed different mechanisms of ITD processing along the auditory pathway. Thompson et al. (2006) presented large ITDs (±1500 μs), well outside the range of ITDs of ±700 μs, that are usually experienced under natural listening conditions. Using functional magnetic resonance imaging (MRI) neural activity was measured by means of the blood oxygenation level dependent response. For these large ITDs, they found higher neural activity in the ipsilateral, compared to the contralateral, side of the mid-brain, which is the opposite of findings for smaller ITDs. A related study by von Kriegstein et al. (2008) revealed that at the level of the cortex, both hemispheres were activated for these large ITDs. For the small ITDs, predominantly the primary auditory cortex in the contralateral hemisphere was active. These data show that coding of ITD in the cortex is fundamentally different from the mid-brain representation of ITD, but it remains unclear how such large ITDs are perceived if lesions impair the encoding or decoding at different stages of the auditory pathway.

Studying clinical populations has shaped our understanding of binaural processing, and is still useful to supplement studies in different animal models (Gallun, 2021). Currently, structural MRI is used in standard clinical routine for stroke patients. The combination of the information on the precise lesion location, and the patients’ performance in behavioral tasks, could lead to insights into individual problems in binaural processing and possible ways to individualize therapies.

The detrimental effects of stroke lesions on binaural hearing tasks vary not only for different lesion locations and lesion sizes, but can also be shaped by factors such as age, conductive or sensorineural hearing loss, cognitive abilities, and other non-auditory characteristics. Therefore, in addition to group analyses that are compared to age-matched control subjects, focusing on individual patients with all their confounding influences case by case remains unavoidable.

The objective of the current exploratory study was to investigate the binaural perception of individuals in the acute phase of stroke, compared to an age-matched control group in a quantitative, yet individual manner. Since binaural deficits have been observed for lesions across multiple brain areas that are not directly related to audition, we did not limit our study to predefined regions of interest. This choice was further motivated by our aim to conduct a relatively large-scale study with potential to reveal patterns that would remain unnoticed or ambiguous with smaller patient cohorts. We conducted two binaural experiments using headphone stimulation. Performance in both experiments relied on using interaural differences. In the first experiment, a binaural tone-in-noise detection task, the implicit use of interaural cues was sufficient to detect differences to the reference stimulus. In the second experiment, a lateralization task, listeners had to explicitly use interaural cues to judge the perceived intracranial position of the stimulus. These experiments, and an additional general assessment, were completed by patients that had rather small lesions in different brain areas. The location of the lesion was established based on previously acquired MRI data.

## 2. Materials and methods

### 2.1 Participants

In total, 50 stroke patients (mean age of 63 years, SD: 14 years, 20 female, 30 male) and 12 control subjects (mean age of 61 years, SD: 14 years, 9 female, 3 male) participated after passing audiometric and cognitive assessments (see Sections “2.2 General assessment” and “2.4.1 Audiometry” for details) and providing written informed consent. Participants that had a stroke will be referred to as patients, whereas those participating in the control group will be referred to as control subjects. The study was approved by the Medical Research Ethics Board of the University of Oldenburg, Germany. The stroke patients were recruited in the stroke unit of the Evangelisches Krankenhaus, Oldenburg, Germany and tested in a quiet room. Only those patients participated who could understand and produce speech, who were mobile and in a general stable condition, and able to complete the different tasks despite their recent stroke. Exclusion criteria were additional neurological diseases or a pure-tone average of 40 dB HL or more (see Section “2.4.1 Audiometry”). The stroke patients participated in the experiments on average 5 days (range: 1—9 days, 16 days for one patient, SD: 2 days) after stroke onset. The symptoms of stroke, as measured by the National Institute of Health stroke scale (see Section “2.2 General assessment”), ranged from 0 to 6 points, except for one patient with a score of 20 points. The median of the scores was one point, thus representing a stroke cohort suffering from minor stroke. The control group was age-matched and followed the same exclusion criteria.

### 2.2 General assessment

Preceding the psychoacoustic experiments, the Montreal Cognitive Assessment (MoCA, Nasreddine et al., 2005) was used to screen for mild cognitive impairment or dementia. The test contains 30 tasks targeting different cognitive abilities, and is scored with a maximum of 30 points. Scores below 26 points suggest mild cognitive impairment. Three patients with a performance score of 17 or lower were excluded from the subsequent experiments.

The National Institute of Health stroke score (National Institute of Neurological Disorders and Stroke [NIHSS], 2019) was obtained as part of the clinical routine 24 h after the patients came to the hospital. It consists of several measures judging the severity of the symptoms of stroke, with a maximum score of 42 points. Scores below 5 are classified as minor stroke, below 15 as moderate stroke, and above this as moderate to severe and severe stroke. The score includes several items related to motor functions, but no item explicitly targeting auditory impairments.

To quantitatively assess the intensity of possible depression, we used the short version of the Beck’s Depression Inventory (BDI, Beck et al., 2013). It contains 7 sets of statements from which are chosen those that best describe the patient’s current state. To be compatible with the full version, the results are scaled to fall within the ranges of the full test. Scores below 9 indicate no or minimal depression, those between 9 and 13 indicate mild depression. Moderate depression is indicated by scores between 20 and 28, and severe depression by scores in the range between 29 and 63.

The multiple-choice vocabulary intelligence test, the German MWT-B (Lehrl, 2005), was used as an estimator for the premorbid intelligence (unaffected by the stroke). It consists of 37 items, each containing five words. Only one of the five words is an established word that must be recognized, whereas the others are neologisms. The higher the number of correctly detected words, the higher the estimated crystallized intelligence (part of a person’s intelligence that consists of knowledge that comes from prior learning and past experiences).

### 2.3 Magnetic resonance imaging

Magnetic Resonance Imaging (MRI) was obtained as part of the clinical routine for all patients. Two different systems were used: a Siemens Magnetom Symphony (1.5 T) and a Magnetom Sola (1.5 T). Lesion location and lesion volume were extracted from these images based on the combined information of diffusion weighted imaging (DWI), apparent diffusion coefficient (ADC) mapping, and the fluid-attenuated inversion recovery (FLAIR) sequence. All areas that were hyperintense in DWI (and had a low signal in the ADC map, thus representing restricted diffusion) were outlined on the FLAIR images using the visualization tool MRIcroGL (Rorden and Brett, 2000), and the volume of the lesions were calculated. The analyses of the images was done using FSL (Jenkinson et al., 2012), a library of analysis tools for FMRI, MRI and DTI brain imaging data. Brain extraction was carried out using FSL BET (Smith, 2002) based on the FLAIR images, since they allowed better extraction than the available T1-weighted images. The fractional intensity threshold for BET was chosen case by case, to obtain the best extraction. The MR images were obtained in the standard clinical routine. Thus, for a majority of patients, only 2D MR images were available. Only in some cases 3D T1 and/or 3D FLAIR data were acquired. Linear registration of the brain-extracted FLAIR images to MNI 152 space, a structural template, provided by the Montreal Neurological Institute, was carried out using FSL FLIRT (Jenkinson et al., 2002). The quality of the resulting images was visually controlled for every subject. The same transformations were applied to the lesion masks. The lesion location was then estimated based on the AICHA atlas (Joliot et al., 2015).

Overlap of the MNI-registered stroke lesions with brain areas that belong to the auditory pathway were estimated as follows: The main nuclei of the primary auditory pathway were defined by the mask provided by Sitek et al. (2019) for the subcortical areas. The auditory cortex was defined by the term-based meta-analyses for the term ‘auditory cortex’ on the website neurosynth.org (Yarkoni et al., 2011), which created a mask using data from 279 MRI studies.

### 2.4 Psychoacoustic measurements

For all of the psychoacoustic experiments, closed headphones with high passive sound attenuation (HDA300, Sennheiser electronic GmbH, Wedemark, Germany) and driven by an external soundcard (UR22mkII, Steinberg Media Technologies GmbH, Hamburg, Germany) were used. The stimuli were generated and reproduced by custom-made MATLAB scripts using the psychophysical measurement package AFC (Ewert, 2013). The sampling rate was 48 kHz.

#### 2.4.1 Audiometry

Pure-tone audiometric testing for a restricted set of frequencies (500, 1000, and 3000 Hz) was conducted preceding the psychoacoustic experiments using a one-interval two-alternative forced-choice procedure controlled by the experimenter. The testing followed a one-up, one-down adaptive procedure. The tracks ended after eight reversals (initial step size was 20 dB, after the second reversal 10 dB, after the fourth reversal 5 dB) and the thresholds were computed from the mean of the last four reversals. The pure-tone average over the three measured frequencies was calculated for the left and right ear individually (PTA3 L and PTA3 R, respectively), and averaged over the two ears (PTA3). In addition, the absolute difference between the left and right PTA3 (PTA3 asymmetry) was calculated. Two patients with a PTA3 L and/or a PTA3 R of more than 40 dB HL were excluded, leading to a total of 50 patients for further study.

#### 2.4.2 Tone-in-noise detection

In the tone-in-noise detection experiments, the participants were presented with three intervals containing 500-ms bursts of octave-wide white noise centered around 500 Hz (333–666 Hz). The stimuli were gated with 20-ms raised cosine onset and offset ramps. The intervals were separated by 300-ms silent gaps. In one of the three intervals, an additional 500-Hz pure-tone of 420 ms duration was added and temporally centered in the noise. The tone had the same ramp parameters as the noise, but its onset was 40 ms later than the noise. Similarly, the tone offset was 40 ms before the noise offset. The participants’ task was to detect the deviating interval (the one containing the tone) and to press key number ‘1,’ ‘2,’ or ‘3’ on a computer keyboard, indicating whether the first, second, or third interval was the odd one. The tone was either interaurally in phase with the noise (condition N_0_S_0_), or had an interaural phase difference of π (condition N_0_S_π_). The experiment started without any training and with two runs of the N_0_S_π_ condition. This was followed by one run of the N_0_S_0_ condition. The noise was presented with 60 dB sound-pressure level (SPL). The level of the tone was initially 65 dB SPL in the N_0_S_0_ condition and 50 dB SPL in the N_0_S_π_ condition. The level varied according to a one-up, three-down procedure, with a step size of 4 dB up to the second reversal, and a step size of 2 dB for the remaining 8 reversals, converging to 79.4% correct thresholds. Thresholds are calculated as the average of the last 8 reversals. If the staircase track hit the maximum tone level of 80 dB SPL during a measurement, re-instructions on how to perform the task were provided. If this did not lead to improvements in task performance, the run was stopped and marked as invalid. No feedback was given during the runs. The binaural masking level difference (BMLD) was calculated from the threshold difference between N_0_S_0_ and the better of the two N_0_S_π_ runs.

#### 2.4.3 Lateralization

For the lateralization task, again a one-octave wide white noise, centered around 500 Hz with an interaural difference in either level or time was presented. The stimuli were generated by copying the same noise sample to both channels and then applying the interaural difference in time or level. The task was to indicate where the sound was perceived inside the head. Responses were given by pressing one of the horizontally aligned numbers ‘1’ to ‘9’ on a computer keyboard, above the letter keys. The participants were instructed to press ‘1’ when the sound was heard on the very left side of their head, ‘5’ for sounds perceived in the center of the head and ‘9’ for the very right side. For possible intracranial positions between the center and the two extremes, the participants were asked to press the respective number ‘2,’ ‘3,’ ‘4,’ ‘6,’ ‘7,’ or ‘8’ on the keyboard. For visual guidance, a template with a schematic drawing of a head indicated the positions of the ears and the center relative to the response buttons. The template covered all of the keyboard except for the numbers ‘1’ to ‘9.’ The duration of the stimuli was 1 s, gated with cosine ramps of 10 ms duration and presented at 70 dB SPL. ITDs ranging from −600 to 600 μs in steps of 200 μs, and two ITDs outside the physiological range (−1500 and 1500 μs), were presented. The ILDs ranged from −12 to 12 dB in steps of 4 dB. The level of the left- and right-ear signals was changed without changing the overall energy by applying the formula presented in Dietz et al. (2013). In addition, monaural stimulation of the left ear and right ear was tested. Each stimulus was presented six times in random order. The diotic stimulus (zero ITD/ILD) was presented eight times. To ensure one common reference system for both types of interaural differences, ILD and ITD stimuli were presented interleaved. In contrast to the investigations by Furst et al. (2000), no training and no center reference were provided in our study. The response to the first trial of each stimulus was not used in further analyses.

Several variables for quantitative description of the lateralization pattern were calculated:

A linear fit to the three left-favoring and right-favoring stimuli, individually for ILD stimuli (−12, −8, −4 dB and 4, 8, and 12 dB) and ITD stimuli (−600, −400, and −200 μs; 200, 400, and 600 μs) was used to describe the steepness of the participants’ lateralization percept (*ILD L slope, ILD R slope, ITD L slope*, and *ITD R slope*). The logarithmic ratio of the left and right slope [*ILD slope ratio, ITD slope ratio, e.g., ILD slope ratio* = *log(ITD slope L/ILD slope R)]* indicates an asymmetric steepness of the two sides.

Variables that inform about side biases in the responses were calculated: The mean of the responses to all ITD or all ILD stimuli (*ITD mean* and *ILD mean*) and the mean of the fit to left-favoring and right-favoring stimuli (*ITD L fit, ITD R fit, ILD L fit*, and *ILD R fit*) were calculated. Furthermore, the mean of those stimuli that were perceived as being in the center of the head (when key ‘5’ was pressed), was calculated for ILD and for ITD stimuli (*ITD center* and *ILD center*). The so-called *diotic percept* was the mean of the responses given for the zero ILD/ITD stimuli.

Another feature of the lateralization data is its variability. For this, the standard deviation for zero ILD/ITD was calculated (*diotic std.*), as well as the mean of the standard deviations of the responses to each ILD stimulus (excluding the monaural stimulation, *ILD std.*), each ITD stimulus (*ITD std.*) and the mean standard deviation of the left-favoring and right-favoring stimuli independently (*ITD L std., ITD R std., ILD L std.*, and *ILD R std.*). Their logarithmic ratios (*ITD std. ratio* and *ILD std. ratio*) can indicate differences in the variability of left-favoring and right-favoring stimuli.

The maximal range of lateralization was calculated by the difference of the maximally lateralized responses given for ITDs within the physiological range (*ITD range*), and for all ILDs excluding monaural stimulation (*ILD range*). The logarithmic ratio of the ranges obtained with ILD and ITD stimuli (*range ratio*) informs about differences in the ranges perceived using the two types of stimuli.

The perception of the monaural left and right (*mon left* and *mon right*), and the ITDs of ±1500 μs (*neg 1500* and *pos 1500*) was only evaluated in terms of the mean response to these stimuli.

For all the variables, values within the interval of 1.5 times the standard deviation around the control group mean were considered to be normal. As we did not want to overemphasize possible asymmetries of the left and right side of individual control subjects, we also added the mirrored control data before calculating the mean and standard deviation. With this, the mean was not biased by individual asymmetries and the standard deviation remained unaffected. We verified that adding the mirrored data did not change the results substantially from those obtained without adding the mirrored responses to the data set. Whenever values of the calculated variables are reported, they are in the unit of response keys (a difference of one response button corresponds to 1/8 of the distance between the two ears), except for the variables describing the goodness of fit and the ratios.

## 3. Results

Analyses will be presented grouped by the presence of stroke (control vs. stroke group) and grouped by the anatomical location of the lesion (lesion groups). In addition, a selected set of individual stroke patients will be shown throughout the results section. These patients are chosen to highlight the individual character of each stroke patient’s performance. The color-coding of the eight selected patients is consistent across Figures, allowing for comparison of their measurement results across experiments. In the last subsection, deviations from the control group are shown for individual cases and for lesion groups.

### 3.1 General assessment

Mean values and standard deviations of the non-auditory testing of the stroke and the control group are shown in Table 1. According to statistical tests (two-sample *t*-tests), the two groups did not differ in age, not in their pure-tone average over the three tested frequencies, and also not in the absolute asymmetry of their left and right PTA3. The scores for the multiple-choice vocabulary intelligence test (MWT-B) and the short form of Beck Depression Inventory (BDI) also did not differ significantly between the groups. In the cognitive screening test (MoCA) however, significantly lower scores were obtained for the stroke group compared to the control group.

**TABLE 1:** General assessment results.

	Stroke *N* = 50	Control *N* = 12	Test statistics
Age [years]	63 (14)	61 (14)	*t*(61) = −0.46, *p* = 0.647
PTA3 [dB HL]	18 (8)	14 (9)	*t*(61) = −1.54, *p* = 0.129
PTA3 asymm. [dB]	4 (4)	5 (4)	*t*(61) = 0.65, *p* = 0.518
MoCA score	23.90 (4.68)	28.36 (1.63)	*t*(60) = 3.10, *p* = 0.003
MWT-B score	29.72 (4.07)	31.37 (4.15)	*t*(59) = 1.21, *p* = 0.231
BDI short score	7.60 (5.04)	6.30 (3.15)	*t*(61) = −0.86, *p* = 0.394

Mean, standard deviation, and t-test results for stroke and control groups. Values are given in the form ‘mean (standard deviation)’.

### 3.2 Audiometry

**FIGURE 1:**
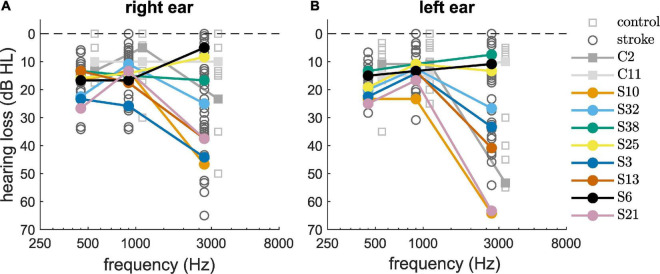
Pure-tone audiometric thresholds of the control group (squares) and the stroke group (circles) measured at the right ear **(A)** and left ear **(B)**. Selected participants are highlighted by the color coding used throughout the figures.

The pure-tone audiometry thresholds for 500, 1000, and 3000 Hz revealed that 35% of the participants had a PTA3 (hearing thresholds averaged over the three frequencies and the two ears) of 20 dB HL or higher, indicating a slight hearing loss (Figure 1). Increased hearing thresholds were especially prevalent at the highest tested frequency of 3000 Hz. Similar PTA3 thresholds were found for the control subjects and for the stroke patients (Table 1), indicating that the pure tone hearing thresholds were not stroke-specific. The selected set of eight stroke patients, indicated by the colored dots, and the two selected control subjects, indicated by the filled gray boxes, span the range of hearing thresholds.

### 3.3 Correlation analyses

We computed correlations between age and PTA3 and the scores obtained from the general assessment (MoCA, BDI, NIHSS, and MWTB). All correlations were computed for the stroke group and the control group together, because the mean values for the two groups did not differ significantly, except for the MoCA scores (see Table 1). The correlation between age and PTA3 was statistically significant (ρ = 0.59, *p* < 0.01). With this, one cannot clearly distinguish between age effects and effects of hearing loss on the other outcome measures. Age and the MoCA score (ρ = −0.36, *p* < 0.01) and PTA3 and the MoCA score (ρ = −0.35, *p* = 0.01) were negatively correlated. The negative correlation between age and the BDI score (ρ = −0.28, *p* = 0.03) was statistically significant, as well. None of the other correlations were statistically significant with the alpha level set to 0.05 (see Table 2 and Supplementary Figure 1).

**TABLE 2:** Correlations between age and PTA3 thresholds and the results of the non-auditory measurements (MoCA, BDI, NIHSS, and MWT-B) for stroke and control group together.

	Age [years]	PTA3 [dB HL]
Age [years]	−	ρ = 0.59, *p* < 0.001
PTA3 [dB HL]	ρ = 0.59, *p* < 0.001	−
MoCA score	ρ = −0.36, *p* < 0.001	ρ = −0.35, *p* = 0.01
BDI score	ρ = −0.28, *p* = 0.03	ρ = 0, *p* = 0.97
NIHSS score	ρ = 0.11, *p* = 0.44	ρ = 0.20, *p* = 0.17
MWT-B score	ρ = 0.14, *p* = 0.29	ρ = −0.18, *p* = 0.17

### 3.4 Tone-in-noise detection

**FIGURE 2:**
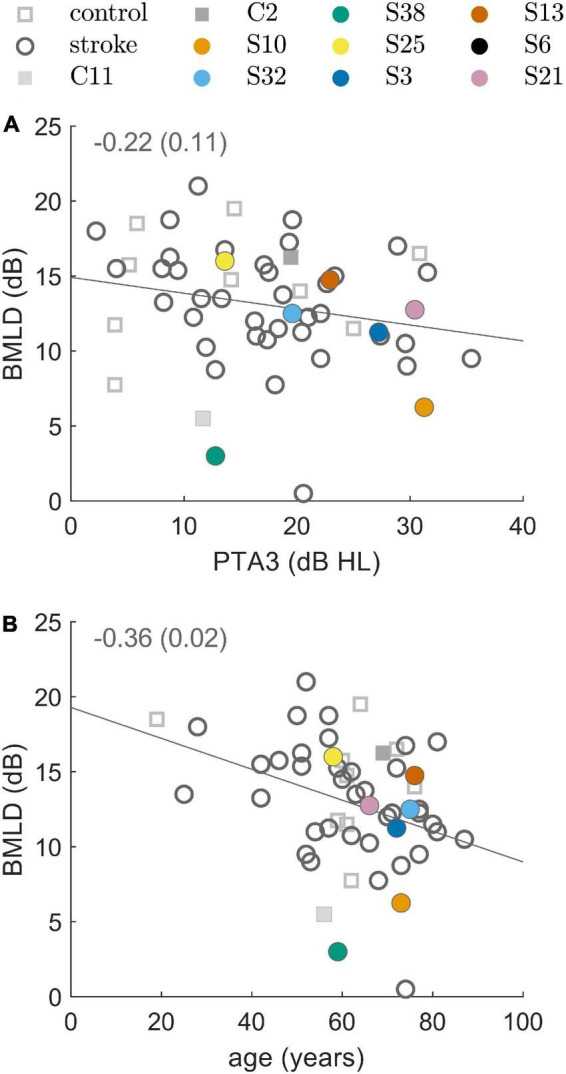
Binaural masking level difference (BMLD) resulting from the binaural tone-in-noise detection experiment. BMLD over PTA3 in panel **(A)** and BMLD over age in panel **(B)** for control subjects (squares) and stroke patients (circles). The line represents a linear fit and the inset represents the correlation coefficient and the respective *p*-value. Selected participants are highlighted by the color coding used throughout the figures.

The majority of participants (44 of the 50 stroke patients, and 11 of the 12 control subjects) produced converging tracks in both conditions of the tone-in-noise detection task. The BMLD was calculated from the difference between N_0_S_π_ and N_0_S_0_ thresholds (see Supplementary Figure 2). Four patients (S6, S18, S20, and S24) and one control subject (C3) produced a convergent track only in the N_0_S_0_-condition, preventing the estimation of the BMLD. S2 and S44 did not produce any converging tracks. It is not known why these participants were not able to perform the task. Due to restricted measurement time, the tasks were not repeated. The normal values of BMLD, as defined by the mean ±1.5 times the standard deviation of the control group results, ranged from 7.5 to 20.1 dB. Of those participants that produced convergent tracks, 93% of the stroke group (41 of 44 patients) showed a BMLD of 7.5 dB or more. This result is comparable to the result from the control group, with 91% of the subjects demonstrating a BMLD of 7.5 dB or more. As shown in Figure 2 there was a significant negative correlation of the BMLD with age (ρ = −0.36, *p* = 0.02), but not with PTA3 (ρ = −0.22, *p* = 0.11).

### 3.5 Lateralization

In general, all participants were able to complete the lateralization task and almost all reported that the monaural stimuli were perceptually different from the binaural stimuli, and that they were the easiest stimuli to lateralize. For many patients, visual inspection of the data did not reveal any impairments in lateralization. Selected group analyses (averages over lesion groups) are presented in Table 3. In the following paragraphs, the observed lateralization patterns of the control group and the lesion groups will be discussed in terms of group averages and examples of individual patients.

**TABLE 3:** Quantification of the lateralization results for the lesion groups.

	*Mean*	*diotic std.*	*ITD range*	*ILD range*	*mon left*	*mon right*
Control (12)	5.2	1.0	5.5	3.7	1.5	8.6
bs l (3)	5.9	1.2	5.3	5.0	1.5	8.7
bs r (4)	4.8	1.4	4.8	4.0	1.3	8.9
thal l (4)	4.7	1.4	4.3	3.4	2.1	8.3
bg l (7)	4.9	1.3	4.8	3.2	1.9	8.7
bg r (4)	5.6	1.6	4.2	2.8	3.1	7.8
multi l (7)	5.3	1.8	5.5	4.4	1.5	7.9
multi r (9)	5.5	1.3	4.8	3.5	2.3	8.5
occi l (3)	5.1	0.8	4.3	4.0	1.1	8.9
cereb l (2)	5.1	1.4	5.1	3.4	1.3	8.5
cereb r (2)	5.7	1.0	3.8	3.2	1.4	8.7
multi b (5)	5.1	2.3	6	4.4	2.0	8.3

Bs, brainstem; thal, thalamus; bg, basal ganglia; multi, multiple lesion sites; occi, occipital lobe; cereb, cerebellum; l, left; r, right. All values are in the unit of response keys (1 = left, 5 = center, and 9 = right).

**FIGURE 3:**
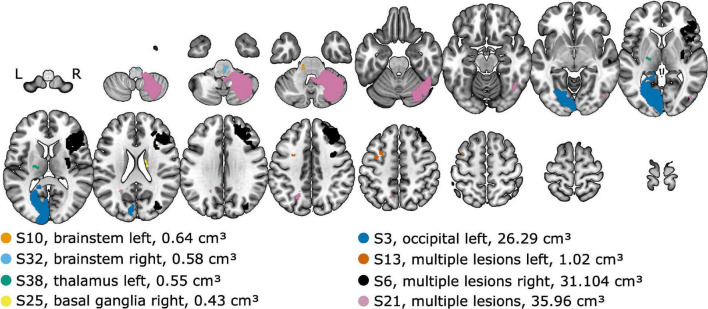
Lesion locations overlaid on axial slices of the MNI152 template. The lesion group and the lesion volume is given in the legend. Selected patients are highlighted by the color coding used throughout the figures.

**FIGURE 4:**
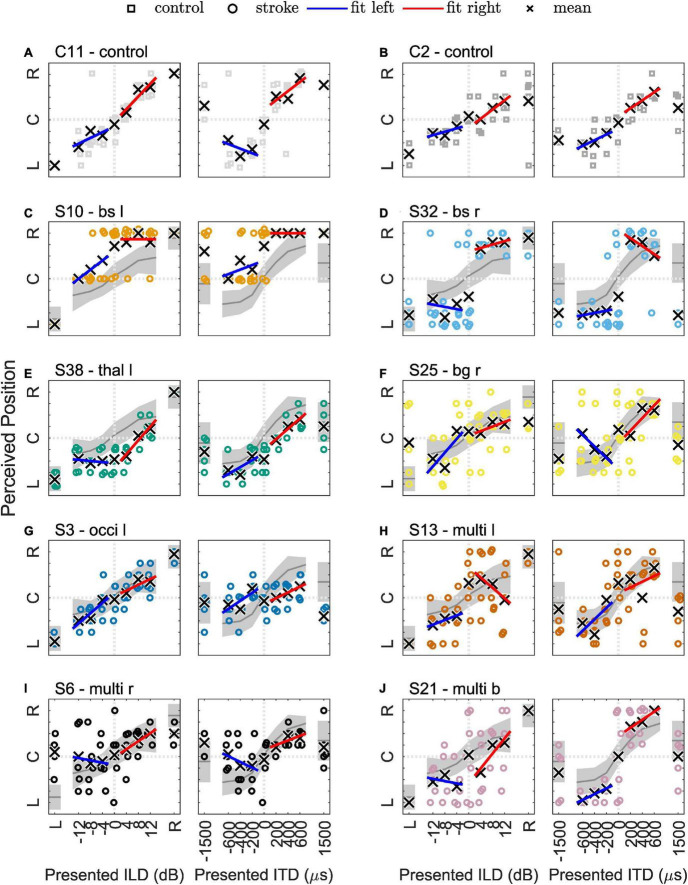
Results of the lateralization task for two example control subjects in panels **(A,B)** and eight selected stroke patients in panels **(C–J)**. The colored symbols represent the responses given to the individual trials of the same stimulus, except for the discarded first trial. The black crosses indicate the means of the given responses. The red and blue lines represent linear fits to right-favoring and left-favoring stimuli, respectively. The gray line and shaded area in panels **(C–J)** indicate the mean and the 1.5 times standard deviation interval around the mean response of the control subjects.

In particular, data from eight patients with different lesion locations and volumes (see Figure 3) were selected for individual presentation. The results of the lateralization task (perceived intracranial position for the presented ILDs and ITDs) are shown in Figure 4 for two example control subjects (panel A and B) and the eight selected stroke patients (panels C-J). These patients are not fully representative of the average patient for their respective lesion group, but rather display distinct response patterns. The lateralization results of all other participants can be found in the Supplementary Figures 3–8.

#### 3.5.1 Control group

Physically left-favoring, to consecutively more right-favoring stimuli, were perceived from the left to the right inside the participants’ heads for the ILD and ITD stimuli for all control subjects, with only slight deviations. Apparently, the chosen ILDs, ranging from −12 to 12 dB did not lead to strongly lateralized auditory images (responses close to response keys 1 = left and 9 = right). Previous studies already demonstrated that the extent of perceived lateralization for ILDs of this magnitude varies across subjects (Baumgärtel and Dietz, 2018). It also depends on frequency, with stronger lateralization perceived for the same ILD magnitude and higher-frequency signals (Bernstein and Trahiotis, 2011). Auditory space was distributed roughly symmetrically around zero ITD/ILD, being reflected in the average perceived position over all ILD and ITD stimuli (*mean*) of 5.2 in the control group. Even in the control group, the perceived intracranial positions were not perfectly distributed around the center (5.0). Monaural left or right stimulation was perceived close to the most lateralized intracranial positions (*mon left*: 1.5 and *mon right*: 8.6) with almost no intra-individual variability. For all ILDs and all absolute ITDs = 600 μs, a small variability in single trials can be seen. The standard deviation of given responses was for all stimuli approximately in the range of one response key for the control subjects (e.g., 1.1 for *diotic std*., the standard deviation of zero ILD/ITD). Only one person of the control group produced much more variable data. The variability of ITDs of ±1500 μs was larger than for smaller ITDs in most control subjects. This unnaturally large ITD was perceived less lateralized compared to smaller absolute ITDs. Based only on the center frequency (500 Hz), one cannot distinguish between a time shift of −500 or +1500 μs, as the period at this frequency is 2000 μs. However, since the stimulus is a white noise of 333 Hz bandwidth centered around 500 Hz, the auditory system can partially resolve this ambiguity, by exploiting either the interaural correlation at other frequencies or the envelope ITD. The *range of lateralization* was larger for ITDs (5.5) compared to ILDs (3.7) and for both interaural differences was much smaller than the maximal possible range of 8.

In the Figures 4A, B, examples of data from two typical control subjects (C2 and C11) show the main trends described above. The colored symbols represent the responses to individual trials of the same stimulus, except for the discarded first trial. The black crosses indicate the means of the given responses. The red and blue lines represent linear fits to right-favoring and left-favoring stimuli, respectively. If no asymmetry was present in a participant’s responses, they should have the same slope on both sides. Completely symmetrical responses to left-favoring and right-favoring stimuli were obtained only by a small number of control subjects. Obviously, for some individual trials the participants’ responses differed from the expected pattern, as for example in one trial with subject C2, the response to monaural-right stimulation was the left-most response key. This intra-individual variability can occur for various reasons. For example, it may be due to perceptual variability *per se*, but could also depend on the state of attention, or lack of concentration when reporting the percept. In Figures 4C–J, the general trends observed in the control group are visualized with the gray line and shaded area indicating the mean and the 1.5 times standard deviation interval around the mean response of the control subjects.

Despite the reduced range of lateralization in most participants, different lesion groups were found to be associated with altered lateralization percepts.

#### 3.5.2 Brainstem lesions

In only one of the seven patients with a brainstem lesion (S42) did the lateralization results visually resemble the control group. All the others showed obvious deviations from the control group. In four of the seven patients of the brainstem lesion group (S7, S10, S12, S22, and S32), a reduced set of response keys was used. The responses were given in the categories left–center–right or only left–right. This is partially reflected in the *diotic std.* of 1.8 for this lesion group. Lesions in the brainstem (medulla, pons, or midbrain) did not alter the perception of monaural stimuli (average of the left-sided and right-sided lesions for the *monaural left* stimulus: 1.4 and 8.8 for *monaural right* stimulation), except in patient S7. Two examples of this group (S10 and S32) are presented in Figure 4 and discussed below.

Patient S10 (73 years) had a lesion in the caudal medulla to rostral pons on the left side. All stimuli, except for the monaural left stimulus, were perceived in the right hemifield (see Figure 4C). This patient gave no responses between center (key 5) and right (key 9). Especially in the case of ITD, right-favoring stimuli were mainly perceived on the right side, whereas left-favoring stimuli were perceived in the center or at the right ear. For the monaural-left stimulus, however, the patient consistently reported the left-most position. The patient had the maximal possible score in the MoCa, but, with a PTA3 of 31 dB, a mild hearing loss and also a PTA3 asymmetry of 11 dB, with a higher threshold in the left ear. The patient was not using a hearing aid. In the tone-in-noise detection task, the track of the binaural condition (N_0_S_π_) was initially approximately 10 dB below the monaural condition (N_0_S_0_). The track finally converged to the monaural threshold, as the interaural information was no longer exploited, leading to a BMLD below the normal range.

A lesion comparable to the case described above, but on the right side, was found in the patient S32 (75 years), and is shown in Figure 4D. The patient never reported a centralized percept (answer keys 4, 5, and 6 were never used) and all stimuli were perceived very close to either ear. The ILD/ITD = 0 stimulus was more often perceived on the left side. Also, both of the supranatural 1.5 ms ITD stimuli were perceived on the left side. This patient had a BMLD of 12.5 dB (within the normal range) and a MoCA score of 22.

Patient S26 (77 years) who was not in the pure-brainstem lesion group, but had multiple lesion sites in both hemispheres, including the left brainstem, also only responded in two categories, but never reported a stimulus to be in the center.

#### 3.5.3 Thalamus lesions

We observed a shift of the auditory space in all patients with a thalamic lesion. However, one left-sided stroke patient showed a shift toward the right side, the other three to the left side. Therefore, the mean responses in this lesion group were only slightly shifted toward the left side (*mean* of 4.7). This also led to a smaller *ITD range* (4.3) and *ILD range* (3.4) than in the control group. In this group, on average, the monaural stimuli were not perceived as much lateralized as in the control group (monaural left: 2.1, monaural right: 8.3). However, this group finding resulted mainly from one patient (S36) that had a high rate of left-right confusions, this was not observed in any other patient of the group.

The patient S38 (59 years) chosen as an example for this group and shown in Figure 4E had a very small lesion in the left lateral thalamus (calculated lesion volume = 278 mm^3^). This damage seems to have led to a shift in the auditory space toward the left side and a reduced range of lateralization. All left-favoring ILD stimuli and the diotic stimulus were perceived at the same position on the right side, indicating that they were indistinguishable by this patient (see Figure 4E). Unlike the other patients with a thalamic stroke, this patient had almost no benefit from binaural listening in the tone-in-noise detection task (BMLD of 3 dB, below the normal range), even though small changes in ITD led to more lateralized percepts. It is unclear, however, if this patient would have improved with more training, as the second run of the N_0_S_π_ condition converged to a lower threshold compared to the first run.

#### 3.5.4 Basal ganglia lesions

Due to the small number of patients in the previously presented groups, comparisons between left-sided and right-sided lesions were not feasible. The basal ganglia lesion group, however, consisted of a larger number of patients (11) with 7 left-sided and 4 right-sided lesions. Comparison of the results between the left- and right-sided lesion cases revealed clear differences in lateralization results. Left-sided basal ganglia lesions resulted in lateralization patterns comparable to the control group. Also, the BMLD for these patients was 10 dB to 19 dB and was thus within the normal range. Patients with right-sided lesions however, showed a higher trial-to-trial variability, compared to the left-sided lesion group. On average, the auditory space of the right-sided stroke group was shifted toward the right side (*mean* of 5.6). Two patients in the right-sided basal ganglia lesion group (S19 and S25) perceived the monaural stimuli more centralized than the control group. One patient (S2) of the right-sided lesion group was not able to carry out the tone-in-noise experiment, while the other three had BMLDs of 11 to 16 dB, within the normal range.

The lateralization results of one of the patients with right-sided basal ganglia damage (patient S25, 58 years) is shown in Figure 4F. In this selected patient, the patterns described above (high variability and shift) are also present. The patient had a BMLD within the normal range (16 dB).

#### 3.5.5 Cerebellar lesions

Four patients had lesions in the cerebellum. By visual inspection, in two of them (S4 and S37) the lateralization performance differed from the control group. In patient S4, with a right-sided lesion, almost no change in lateralization for different ITDs could be observed, but a smooth, though flat, transition for ILD-based lateralization. Patient S37 showed no impairments in ITD-based lateralization, but the variability of left-favoring ILD stimuli was larger than for right-favoring ILD stimuli. All BMLDs of this group were within the normal range.

#### 3.5.6 Multiple lesions in one hemisphere

In many cases, stroke lesions were distributed over several cortical and subcortical areas (see, e.g., patient S6 in Figure 3). Therefore, this group is especially heterogeneous. Almost all patterns described in the previous groups can be found in some of the patients in this group. In more than half of the cases, large differences to the control group can be observed by visual inspection. The trial-to-trial variability of the given responses was increased in a large number of patients with multiple cortical lesions, even if the auditory cortex was not directly affected (e.g., S23 and S48). Especially contralesional difficulties, as shown by highly variable lateralization responses or a less steep slope in the contralesional hemifield, can be found (e.g., S13 and S20). Interestingly, only in two patients (S6 and S48) was a neglect reported with the NIHSS tests. Both had increased variability on the left (contralesional) side and reported some of the left-favoring stimuli on the right side. For some patients (e.g., S6, S20, and S41) with right-sided cortical and subcortical lesions, the left-favoring and the right-favoring stimuli with an ITD of ±1500 μs were both perceived on the right (the ipsilesional) side. With multiple lesion sites in the left hemisphere, only one patient (S13) had this ipsilesional shift, whereas two others (S29 and S45) also had a shift toward the right—in this case contralesional side.

Two of three patients with damage to the occipital lobe showed almost normal patterns of lateralization, and BMLDs of 11 dB and 18 dB (within the normal range). The third member of this lesion group (patient S3, 72 years, lateralization results shown in Figure 4G) showed almost no sensitivity to ITD-based stimuli, whereas ILDs led to lateralization within the normal range, very similar to the cerebellar stroke patient S4 described above. Compared to the other group members, patient S3 had a slightly reduced BMLD of 8 dB, just within the normal range.

In patient S13 (76 years, presented in Figure 4H) damage mainly to the superior frontal lobe on the left side led to an almost normal lateralization performance in the ipsilesional hemifield, but increased variability for the zero ILD/ITD stimulus and right-favoring stimuli. The monaural left and right stimuli and the BMLD were unremarkable and within the normal range.

Patient S6 (62 years) had widespread lesions in the right hemisphere, including temporal and frontal cortex areas, the insula and basal ganglia. This patient showed high variability for the left monaural stimulus, whereas responses to the right monaural stimulus did not vary much from trial to trial (Figure 4I). In this patient, the difference between the left and the right hemifield was even stronger than in S13. The responses to right-favoring interaural differences varied very little, whereas the left-sided (contralesional) stimuli varied a lot and were even sometimes reported on the other side. This person also showed signs of neglect that were captured with the NIHSS. Again, the BMLD was not affected.

#### 3.5.7 Multiple lesions in both hemispheres

Compared to the other lesion groups, data interpretation for the patients with multiple lesions distributed in both hemispheres was very difficult. None of these patients showed results similar to the control group.

Patient S21 (66 years), presented in Figure 4J, had small lesions in the left precuneus cortex and the right occipital cortex, but a large lesion in the right cerebellum, probably also including small portions of the left medulla. This patient had a NIHSS score of 20 points, but the item on neglect was rated with zero points. Patient S21 showed considerable differences in lateralizing ILD- or ITD-based stimuli. ITD-based lateralization appeared mostly unaffected, whereas the ILD-based lateralization results were shifted toward the left with high trial-to-trial variability. However, this could also be related to the PTA3 difference of 9 dB between the two ears (right ear more sensitive). Nevertheless, responses to stimuli without any interaural difference varied strongly, but adding an ITD of 200 μs or −200 μs already led to strong and reliable right-lateralized or left-lateralized percepts. The BMLD of 13 dB was within the normal range.

#### 3.5.8 Lesions on the primary auditory pathway

This lesion group contains patients for which the MNI-registered lesion outline overlaps to some extent with areas of the primary auditory pathway (subcortically or cortically). These patients are already included in the previous lesion groups. Many patients of this group show distinct lateralization patterns. Three of the selected subjects shown in Figure 4 had lesions of the auditory pathway. S10 (Figure 4C) had a lesion of the left superior olivary complex (SOC), S32 (Figure 4D) a lesion of the right SOC, and S6 (Figure 4I) a lesion of the right auditory cortex. Altered lateralization patterns were also found for S7 (lesion close to the left cochlear nucleus and SOC) and S31 (lesion between SOC and inferior colliculus). This indicates that direct involvement of the auditory pathway does affect the lateralization in almost all cases. However, in S14 (multiple lesions close to the left SOC and dorsal of the right AC) and S16 (partial overlap with left AC) parts of the auditory pathway seem to be affected without leading to obvious influences on these patients’ lateralization performance.

### 3.6 Differences to the control group

**FIGURE 5:**
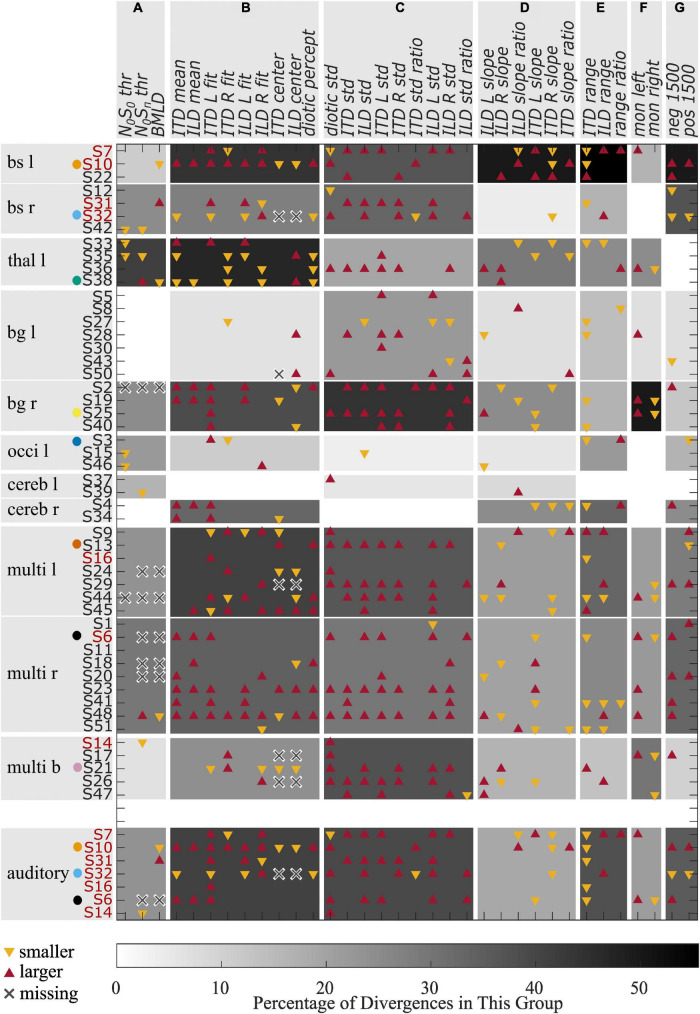
Divergences from the control group for different variables. Red upward triangles indicate values of individuals that are larger and yellow downward triangles values that are smaller than the normal values (mean ± 1.5 times standard deviation) calculated from the control group. Crosses indicate missing values. The gray shading indicates the percentage of deviations found within one lesion group for one of the variable groups **(A–G)**. The red font is used for those patients who had a lesion on the primary auditory pathway. Selected patients are highlighted by the color coding used throughout the figures.

Verbal description of the performance in the two experiments as given above fails to reveal some of the general patterns within specific lesion groups. An attempt to quantify the results of both experiments relative to the control group is shown in Figure 5, showing divergences from the control group for all individual patients for different variables calculated from the results of the tone-in-noise detection experiment and the lateralization experiment. For each variable, the upward and downward triangles indicate higher or lower values compared to the normal range (mean ± 1.5 standard deviation) of the control group. The patients are grouped according to the lesion locations. The variables are clustered in group *A* to group *G*, describing different response characteristics. The gray shadings indicate the percentage of deviations from the control results within each specific subgroup (lesion group and variable cluster). For the lesion group ‘brainstem left’ for example, the percentage of divergences in cluster *A* is approximately 11 percent (one out of nine).

The variables in cluster *A* are the thresholds of the tone-in-noise experiment. For these variables, the strongest divergences were found in the ‘thalamus left’ lesion group. Cluster *B* consists of variables describing a shift of auditory space. Again, the ‘thalamus left’ lesion group showed the most divergences for these variables, followed by the groups ‘brainstem left,’ ‘multiple lesions left,’ and ‘basal ganglia right.’ The highest percentage of divergences in cluster *C* (variability of the data) can be observed for the ‘basal ganglia right’ group, followed by the groups ‘brainstem left,’ ‘multiple lesions bilateral,’ and ‘multiple lesions left.’ The highest percentage of divergences from the control group in variables of cluster *D* are found in the ‘brainstem left’ lesion group. Cluster *D* is a collection of variables that describe the slopes of the fits. Cluster *E* describes the ranges of ITD- and ILD-based stimuli, as well as the difference between the ranges. Again, the most divergences are found for the group ‘brainstem left.’ The perception of monaural stimuli (cluster *F*) differed from the control group most for the lesion group ‘basal ganglia right,’ whereas the large ITDs outside the physiological range (cluster *G*) were perceived differently to the control group by the groups ‘brainstem left’ and ‘brainstem right.’

From the data presented in Figure 5, it becomes apparent that lesions in the left basal ganglia, the occipital lobe and the cerebellum did not lead to lateralization patterns that differ from the control group to any great extent (no more than 33 percent), whereas divergences in many variable clusters are found for patients with damage in the brainstem, the thalamus, and right basal ganglia, and for those individuals with multiple lesions in one or both hemispheres. Much stronger differences, especially in clusters *B, C*, and *F* are present in those patients with lesions to the right basal ganglia compared to left basal ganglia. Furthermore, all but one of the seven patients who were not able to complete the tone-in-noise detection experiment had multiple lesion sites.

**FIGURE 6:**
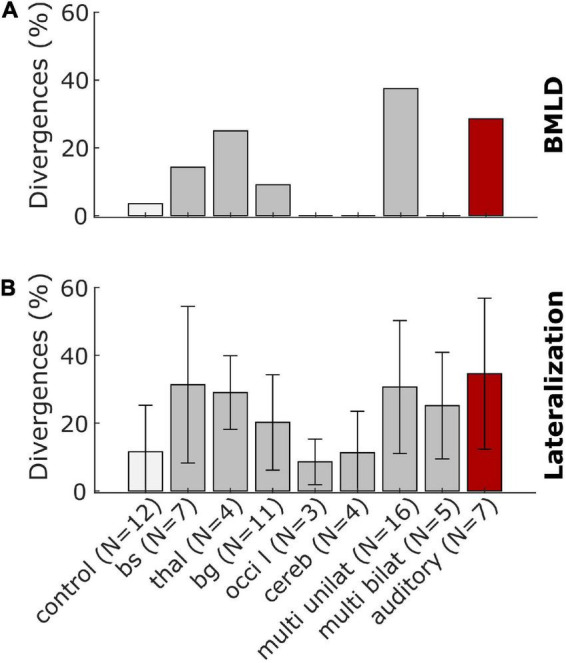
Summary representations of the stroke-related binaural hearing deficits across patient groups. Panel **(A)** shows the percentage of patients who had BMLD-values that were worse (*smaller*) than the control group mean by more than 1.5 standard deviations or could not complete the task. Panel **(B)** shows the percentage of possible divergences of all values calculated for the lateralization patterns with error bars denoting the standard deviation across participants for the control and lesion groups.

The data presented in Figure 5 was condensed to a simpler representation by extracting the percentage of divergences of the BMLD and a general measure of lateralization performance by averaging over the number of divergences of all variables in clusters *B-G*. Lesion groups were pooled over left-sided and right-sided groups. The value for these simplified BMLD and lateralization measures are shown in Figures 6A, B, respectively. Note, that panel A represents the percentage of patients showing smaller than normal or non-convergent tracks in the BMLD task, whereas panel B represents the mean percentage of possible deviations in a given group with the error bars denoting the standard deviation across participants in the group.

For two of eleven patients with lesions in the brainstem or the thalamus, the BMLD diverged from the normal values. One patient of each lesion group had a BMLD of less than 7.5 dB. One patient with a lesion of the basal ganglia and five of 16 patients with unilateral cortical lesions did not produce converging tracks in the task, representing the most remarkable divergence in this task. No divergences were observed for the other lesion groups (see Figure 6A). Two of seven patients with a lesion on the primary auditory pathway diverged from the normal values. One of these two patients produced a BMLD of 6.25 dB, the other one did not produce converging tracks in the dichotic condition of the task. In general, deviations of the BMLD were not frequently observed in the stroke group.

In contrast, for all lesion groups, divergences in terms of the lateralization pattern are found (see Figure 6B). Both measures have the highest percentage of divergences for the patients with a lesion on the primary auditory pathway as shown with the red bars in Figure 6.

## 4. Discussion

In the present exploratory study, our aim was to investigate the binaural perception of individuals in the acute phase of stroke. The performance of the stroke patients in two binaural headphone experiments and the results of the general assessment were compared to an age-matched control group. To our knowledge, this was the first time that the same binaural hearing tasks were conducted in acute-phase stroke patients with various lesions, ranging from the brainstem up to cortical areas. Interpreting these data is a challenging endeavor, especially for the results of the lateralization task, where several metrics are possible and necessary. Using various approaches of comparing patients on a group level and individually with the control group, we found impaired binaural hearing in the majority of stroke patients as shown in Figures 5, 6.

One of the most prominent results was that some of the brainstem-lesion patients lateralized ITD and ILD stimuli in a categorical manner, as suggested by the fact that only a reduced set of response keys was used. For instance, some of these patients commonly gave responses in the categories left-center-right or only left-right, with no responses at intermediate positions. As the information from the left and right ear is integrated in brainstem nuclei for the first time, strongly altered lateralization patterns were expected for the patients who had suffered a stroke to these structures. Accordingly, some of the most prominent distortions in spatial perception were found for brainstem lesion patients. For instance, the cases without responses in the center position were almost exclusively associated with damage of the brainstem (e.g., S32). For this lateralization pattern, at least two interpretations are possible. First, it is possible that a fused image was perceived, but it was lateralized very much toward the sides. An alternative explanation would be that binaural fusion failed for these subjects. As a result, they might have perceived split auditory images (two separate sound sources rather than a single fused image) and reported the position of the dominant image. This ambiguity could be resolved by asking for the number of perceived sound sources in any subsequent studies. The described pattern of side-oriented lateralization was also reported by Furst et al. (2000) for lesions in rostral parts of the brainstem. In contrast to their findings, we did not observe center-oriented patterns (consistently no lateralized percept) in any of the patients from the brainstem lesion group. Despite the many differences between the brainstem patients and the control group as seen in Figure 5, both ILD and ITD stimuli evoked lateralized percepts in all but one patient (S31) of this lesion group. The mean responses of this patient were close to center for all ILD stimuli and left-sided ITD stimuli. The patient had a reduced ITD range, but also a larger standard deviation than the control group. This pattern of responses is suggestive of reduced sensitivity to interaural cues rather than of a center bias.

Left-sided thalamic lesions were, in all cases, correlated with a shift in the lateralization results for both ILD and ITD stimuli. This becomes clear from the high prevalence of deviations in cluster *B* of this group shown in Figure 5. Three out of four patients of this lesion group showed a shift toward the ipsilesional side. No conclusion on the effects of left- vs. right-sided lesions can be drawn, because none of the patients in this study had a lesion of the right thalamus. In addition, one subject with a thalamic lesion displayed remarkably high trial-to-trial variability in their lateralization responses. The medial geniculate nucleus (MGN) located in the auditory thalamus receives projections from the ipsilateral and contralateral inferior colliculus and projects to the ipsilateral auditory cortex (Pickles, 2013). Assuming that these projections are damaged by the stroke, one possible explanation for the lateralization shifts could be that corrupted inputs reach the MGN. Higher variability could be related to altered inputs to the cortical representation stages (outputs of the MGN). Besides damage to auditory nuclei, shifts in auditory space could also be related to asymmetrical hearing thresholds (Florentine, 1976). The hearing thresholds at 500 Hz were symmetrical between the two ears in all patients of this lesion group (except for the one with the increased trial-to-trial variability), but the PTA3 asymmetry was in a range of 4 to 13 dB and pointed toward the direction of the shift. This is in agreement with a significant correlation of PTA3 asymmetry with both ILD mean (ρ = 0.34, *p* = 0.017) and ITD mean (ρ = 0.34, *p* = 0.016) when including all patients of the stroke group. Even though the PTA3 asymmetry can influence the results of the lateralization task, the finding of shifted auditory space for all thalamic-lesion patients indicates an influence of the left thalamus on lateralization. This is in line with previous studies that have found a connection between thalamus lesions and visuospatial neglect (Karnath et al., 2002).

A biased auditory egocentric space in cases with inferior parietal and frontal dysfunction was reported by Bellmann et al. (2001). Further, they found an imbalance of attentional load allocated to the left and right hemispaces (hemispatial inattention) following lesions of basal ganglia and insular cortex. Both mechanisms (biased spatial perception and unbalanced spatial attention across hemifields) come into play for our lateralization task, but their effects are difficult to distinguish in our data. Shifted auditory space and altered lateralization slopes (steepness of the lateralization function of ITD/ILD, see, e.g., S38) indicate distortions of spatial representation. Increased trial-to-trial variability, on the other hand (e.g., S13 and S25), may be indicative of attentional or cognitive impairments, or both. Also, Gutschalk and Dykstra (2015) concluded that more work is needed to develop clinical protocols that can clearly distinguish localization deficits from disorders of spatial cognition. The effects of the right basal ganglia on the lateralization patterns that we observed, could be attributed to attentional deficits. In contrast to Bellmann et al. (2001), our results show that the perception of both left-favoring, as well as right-favoring stimuli was affected in some patients (see Figure 5). Given the supra-modal nature of the neglect syndrome, a basal ganglia lesion may affect auditory and visual hemispatial attention. Influences of right basal ganglia lesions on the visuospatial perception of both, ipsi- and more frequently contralesional stimuli were already reported by Karnath et al. (2002).

For almost all patients with multiple lesions in one or both hemispheres, we found lateralization patterns that differed from the control group in terms of increased variability and decreased slopes, as shown by the high number of divergences in the clusters *C* and *D* of Figure 5. Besides contralesional deficits as in patient S6 with multiple lesion sites, including the right temporal lobe, many patients also displayed ipsilesional deficits for both left- and right-sided lesions. This is only partially in line with previous literature (see Häusler and Levine, 2000 for a review) that suggests a dominance of the right hemisphere in auditory spatial representation. In our study, a comparison of left-sided and right-sided cortical lesions might not be meaningful, because of the unequal distribution of lesion sites. Since the inability to understand and produce speech is mainly observed after damage to left-hemispheric language areas, and was one of the exclusion criteria, left-sided and right-sided groups differed in terms of their lesion locations. For basal ganglia lesions however, strong differences between the left and right side were observed, with more frequent and more severe deficits after right-sided lesions than for left-sided lesions. This result is similar to the results presented in Karnath et al. (2002) for the visual modality.

The perception of ±1500 μs ITDs, i.e., ones that are larger than those usually experienced under natural listening conditions, was only rarely affected. In the brainstem-lesion patients S10, S22, and S32, the left-favoring and right-favoring stimuli were both perceived on the contralesional side. The ambiguity of this stimulus stems from the conflicting interaural cues conveyed by the envelope (indicating the position on the leading side) and the temporal fine structure (indicating a stimulus on the opposite site). With damage in one side of the brainstem, the ipsilesional cue may not be accessible to the next processing stage or less weight might be given to it. With multiple cortical and subcortical lesions, the outcomes are more diverse. While some patients (e.g., S13 and S20) perceived both of these stimuli only on the ipsilesional side, other patients (e.g., S29 and S45) perceived them exclusively on the contralesional side. These findings point to the interpretation that disturbances at different levels of ITD representation stages can lead to stimuli with unnaturally large ITDs being perceived at different intracranial positions. Coding of such large ITDs was already found to differ at midbrain, compared to cortical, levels (Thompson et al., 2006; Kriegstein et al., 2008). While the exact combination of computational processes by which the auditory system encodes ITDs remains elusive, stroke lesion studies such as the present one could potentially aid in their elucidation. However, due to the rarity of psychoacoustic data from stroke survivors, combined with the highly individual nature of stroke lesions, more data is needed before meaningful interpretations are possible.

The dichotic tone-in-noise detection task is a better test for the implicit use of interaural differences compared to the more commonly used measurements of just-noticeable differences in ILD and ITD cues. In many cases, the performance in these tasks depends on the explicit perception of intracranial positions rather than on the general ability to exploit binaural cues for unmasking. To be able to directly compare the results of the implicit tone-in-noise detection task with those of the explicit lateralization task, we refrained from using speech-related tasks such as the one used in, e.g., Tissieres et al. (2019). The results of our lateralization task revealed that five of the six patients with lesions in the right basal ganglia showed remarkable impairments in ITD-based lateralization, which requires the explicit use of interaural differences. Four of these five patients had a BMLD in the normal range (and one only slightly below the normal range), indicating that they had access to implicit ITD information, despite the fact that they could not exploit ITDs explicitly in the lateralization task. This reveals that altered ITD-based lateralization is not necessarily related to dysfunctional encoding at the primary stage in the superior olivary complex. Instead, it seems that damage to the explicit representation stages can impair lateralization even if the primary encoding stages remain unaffected. In general, few patients had smaller than normal BMLDs. Similarly, also Lynn et al. (1981) reported that the speech BMLD was not affected in patients with lesions on cerebral, thalamic, midbrain or rostral pontine levels. In their study, only patients with lesions at the ponto-medullary level showed a reduced BMLD. In our study, two patients had a lesion at the ponto-medullary junction. One of these two patients had a reduced BMLD. Only two of the remaining 48 patients with lesions at other areas had a reduced BMLD. Due to these low numbers, no clear supporting or contradicting conclusions can be drawn. On the other hand, the inability to do the tone-in-noise detection task (missing values due to non-convergent tracks, indicated by crosses in Figure 5) was observed in some patients in which, among other areas, the basal ganglia were damaged and in some patients also frontal cortical areas. Cortico-striatal loops have been shown to be involved in auditory discrimination learning (Znamenskiy and Zador, 2013), which is a necessary ability for this experiment. This implies that the slightly more complex tone-in-noise detection task needs to be learned first, and may therefore not be an optimal measure of the accessibility of implicit interaural information for participants with learning difficulties. Besides the theoretical implications, the deviations in the BMLD as shown in Figure 6, and in particular the inability to complete the task, could be of clinical interest. The BMLD is correlated with age, but the occurrence of stroke does appear to constitute an additional factor affecting binaural tone-in-noise detection performance for some stroke patients. As such, the BMLD could potentially be used clinically to detect effects of stroke on binaural hearing.

Due to the heterogeneous group of participants and the highly individual nature of stroke lesions, the present study is affected by a number of confounding factors. We sought to capture some of these by additional auditory and non-auditory measures such as the audiometry and the MoCA. To paraphrase Gallun (2021), the perturbations caused by nature and not manipulated in the laboratory are never uniform and not easily documented.

In the present study, the selection of patients could not control for the influence of age and hearing loss, but the control group was age-matched and did not differ significantly in their hearing thresholds or in the results of the general assessment. Only the results of the MoCA differed significantly between the stroke and control groups (see Table 1). Almost all non-stroke-related difficulties should be rather equally present in both groups. We therefore concluded that the observed effects on a group level, though not on an individual level, can be attributed to the stroke and possible comorbidities, rather than on hearing loss. The selection of those cases presented in Figures 3, 4 was based on the results of the lateralization task. The selected stroke patients span the whole range for all measured variables (see Supplementary Figure 1). For the stroke patients, of course, the premorbid performance is not known.

The stimuli of both experiments were chosen to be centered around 500 Hz, which is usually spared by age-related hearing loss. The threshold for this frequency was on average 16 dB HL and did not exceed 35 dB HL for any participant. No more than a 10 dB difference between the left and right side was measured at this frequency for any of the participants. We therefore did not expect large influences of hearing loss or asymmetrical hearing abilities on our results. Nevertheless, as discussed above, a correlation of PTA3 asymmetry and shifted auditory space was found.

We focused only on those lesions that had a high signal on the DWI and a low signal in the ADC map, thus representing restricted diffusion. In many cases, older lesions and other damage to brain tissue were present that could have influenced performance in the different tasks. However, improvements from diaschisis or functional reorganization is known to drive neurologic recovery already in the acute phase (Sang-Bae and Byung-Woo, 2013). In addition, in healthy subjects, reorganization of lateralization with altered ITD cues occurs within few days (Trapeau and Schönwiesner, 2015). This suggests, that binaural hearing impairments are dominated by the acute damage and less by old lesions. Complete lesions of specific parts of the brain are used to study the system in ablation studies in animals. In our patients however, the damage does not necessarily include entire brain structures and may leave some functioning neuronal processing. Furthermore, as pointed out by Neff et al. (1975), experiments in well controlled ablation studies in animals measure the functioning of the remaining system and not necessarily the functioning of the damaged part. In contrast to such ablation studies, the general state of brain structures that were not damaged by the acute stroke varied widely in our population. The observed variability in performance must therefore be partially attributed to differences in the damages as well as to differences in the remaining brain structures, rather than solely to the acute stroke lesion.

Not only did individual characteristics of the patients affect the data, but also external constraints such as the restricted time for the behavioral experiments. The short time we had with the patients did not allow for dedicated training runs nor for repetitions of any task. One example where more time would have been necessary was when patients were not able to do the tone-in-noise experiment. In retrospect, from the trend in these patients’ adaptive tracks, it appeared as if some of these patients would have learned to do the task had there been more runs of the same experiment. In addition, the hospital room in which the study was conducted was comparably quiet, but had no sound booth. Finally, the fact that some lesion groups contained only two patients, allowed only limited interpretations. Differentiation between the effects of lesions of a particular anatomical structure as opposed to differences between left-sided and right-sided lesions of that brain area is restricted.

From the data obtained in our experiments, we do not know if these patients also had difficulties in free-field-localization tasks, in which spectral cues are available in addition to natural combinations of ILDs and ITDs. However, as both cues are often perceived with a similar bias and spectral cues are less salient in elderly listeners, we assume that some patients will have localization biases, at least during the acute phase. If a bias remains in the chronic phase of stroke, individualized ILD- and ITD-manipulating algorithms could potentially be exploited to improve localization performance (e.g., Brown, 2018).

## 5. Conclusion

This exploratory study revealed some expected divergences in binaural perception between the results of patients with acute ischemic stroke lesions and the results of the control group: Impaired contralesional lateralization was found after right cortical and brainstem lesions, which is consistent with previous reports. Other findings could be expected, based on today’s understanding of binaural processing and decoding of spatial cues: The perception of binaural stimuli with unnaturally large ITDs is affected differently based on the lesion location. Other findings were less expected, such as the shift in auditory space in all patients with thalamic lesions or the large difference induced by left and right basal ganglia lesions. In contrast to previous reports, no apparent hemispheric difference from cortical lesions regarding the variability of lateralization data were found, and the binaural benefit in the tone-in-noise detection task was unaffected in most patients, although many patients with multiple lesion sites could not complete this task. While it may be too early to suggest any revisions to our understanding of interaural cue encoding or decoding, the outcomes may nevertheless foster more focused future investigations in selected groups of patients with specific lesions, or in animal models. Investigating acute-phase stroke patients may even be an additional avenue to deepen our understanding of the healthy auditory system in a way that is difficult when studying the healthy system in isolation.

## Data availability statement

The dataset analyzed for this study can be found at https://doi.org/10.5281/zenodo.7415436. Due to ethical restrictions, only the lesion masks, but no raw MRI images, are provided. Further inquiries can be directed to the corresponding author.

## Ethics statement

The studies involving human participants were reviewed and approved by the Medical Research Ethics Board of the University of Oldenburg, Germany. The participants provided their written informed consent to participate in this study. Written informed consent was obtained from the individual(s) for the publication of any potentially identifiable images or data included in this article.

## Author contributions

AD, MD, and PS contributed to the conception and design of the study. AD, MD, PS, and KW planned the experimental procedures. AD organized the data base, performed the analysis, and wrote the first draft of the manuscript. MB and AM recruited participants and acquired data. AD, PS, and BS did MRI analyses. AD and MD interpreted the data. MD and HP wrote further sections of the manuscript. All authors contributed to manuscript revision, read, and approved the submitted version.

## Supplementary material

A figure with the correlations between age and PTA and the general assessment scores and the tone-in-noise-detection thresholds, and the lateralization results, such as those presented in Figure 4 for all control subjects and all stroke patients, are available as supplementary data.

Supplementary Figure 1 Scatter plots representing correlations between age and PTA3 thresholds and the results of the non-auditory measurements (MoCA, NIHSS, BDI, and MWT-B) for the control group (squares) and the stroke group (circles). In each subpanel, linear-regression lines, the Pearson correlation coefficient ρ, and the respective *p*-value are shown in the form “ρ (*p*-value)”. Selected participants are highlighted by the color coding used throughout the figures.

Supplementary Figure 2 Results of the binaural tone-in-noise detection experiment. Tone-in-noise detection thresholds for N0S_π_ condition (up- and downward triangles for stroke and control subjects, respectively) and N0S0 condition (left- and right-pointing triangles) over PTA3 **(panel A)** and over age **(panel B)**. In each subpanel, linear-regression lines, the Pearson correlation coefficient ρ, and the respective *p*-value are shown in the form “ρ (*p*-value)”. Selected participants are highlighted by the color coding used throughout the figures.

Supplementary Figure 3 Results of the lateralization task for patients S1–S10. The circles represent the responses given to the individual trials of the same stimulus, except for the discarded first trial. The black crosses indicate the means of the given responses. The red and blue lines represent linear fits to right-favoring and left-favoring stimuli, respectively. The gray line and shaded area indicate the mean and the 1.5 times standard deviation interval around the mean response of the control subjects.

Supplementary Figure 4 Results of the lateralization task for patients S11–S20. The circles represent the responses given to the individual trials of the same stimulus, except for the discarded first trial. The black crosses indicate the means of the given responses. The red and blue lines represent linear fits to right-favoring and left-favoring stimuli, respectively. The gray line and shaded area indicate the mean and the 1.5 times standard deviation interval around the mean response of the control subjects.

Supplementary Figure 5 Results of the lateralization task for patients S21–S30. The circles represent the responses given to the individual trials of the same stimulus, except for the discarded first trial. The black crosses indicate the means of the given responses. The red and blue lines represent linear fits to right-favoring and left-favoring stimuli, respectively. The gray line and shaded area indicate the mean and the 1.5 times standard deviation interval around the mean response of the control subjects.

Supplementary Figure 6 Results of the lateralization task for patients S31–S40. The circles represent the responses given to the individual trials of the same stimulus, except for the discarded first trial. The black crosses indicate the means of the given responses. The red and blue lines represent linear fits to right-favoring and left-favoring stimuli, respectively. The gray line and shaded area indicate the mean and the 1.5 times standard deviation interval around the mean response of the control subjects.

Supplementary Figure 7 Results of the lateralization task for patients S41–S50. The circles represent the responses given to the individual trials of the same stimulus, except for the discarded first trial. The black crosses indicate the means of the given responses. The red and blue lines represent linear fits to right-favoring and left-favoring stimuli, respectively. The gray line and shaded area indicate the mean and the 1.5 times standard deviation interval around the mean response of the control subjects.

Supplementary Figure 8 Results of the lateralization task for control subjects C1–C12. The squares represent the responses given to the individual trials of the same stimulus, except for the discarded first trial. The black crosses indicate the means of the given responses. The red and blue lines represent linear fits to right-favoring and left-favoring stimuli, respectively.

## References

[B1] Bamiou D.-E. Werring D. Cox K. Stevens J. Musiek F. E. Brown M. M. (2012). Patient-reported auditory functions after stroke of the central auditory pathway. *Stroke* 43 1285–1289. 10.1161/STROKEAHA.111.644039 22382162

[B2] Baumgärtel R. M. Dietz M. (2018). Extent of sound image lateralization: Influence of measurement method. *Acta Acust. United Acust.* 104 748–752. 10.3813/AAA.919215

[B3] Beck A. T. Steer R. A. Brown G. K. (2013). *Beck depressions-inventar - FS (BDI-FS): Deutsche bearbeitung.* London: Pearson.

[B4] Bellmann A. Meuli R. Clarke S. (2001). Two types of auditory neglect. *Brain* 124 676–687. 11287368 10.1093/brain/124.4.676

[B5] Bernstein L. R. Trahiotis C. (2011). Lateralization produced by interaural intensitive disparities appears to be larger for high- vs low-frequency stimuli. *J. Acoust. Soc. Am.* 129 15–20. 10.1121/1.3528756 21302976 PMC3037973

[B6] Brown C. A. (2018). Corrective binaural processing for bilateral cochlear implant patients. *PLoS One* 13:e0187965. 10.1371/journal.pone.0187965 29351279 PMC5774684

[B7] Dietz M. Bernstein L. R. Trahiotis C. Ewert S. D. Hohmann V. (2013). The effect of overall level on sensitivity to interaural differences of time and level at high frequencies. *J. Acoust. Soc. Am.* 134 494–502. 10.1121/1.4807827 23862824 PMC3724750

[B8] Ewert S. (2013). “AFC - A modular framework for running psychoacoustic experiments and computational perception models,” in *Proceedings of the international conference on acoustics AIA-DAGA*, Merano, 1326–1329.

[B9] Florentine M. (1976). Relation between Lateralization and loudness in asymmetrical hearing losses. *J. Am. Audiol. Soc.* 1 243–251. 931759

[B10] Furst M. Aharonson V. Levine R. Fullerton B. Tadmor R. Pratt H. (2000). Sound lateralization and interaural discrimination. Effects of brainstem infarcts and multiple sclerosis lesions. *Hear Res.* 143 29–42. 10.1016/s0378-5955(00)00019-8 10771182

[B11] Gallun F. J. (2021). Impaired binaural hearing in adults: A selected review of the literature. *Front. Neurosci.* 15:610957. 10.3389/fnins.2021.610957 33815037 PMC8017161

[B12] Gatehouse (2004). *The speech, spatial and qualities of hearing scale (SSQ).* Perinton, NY: Gatehouse. 10.1080/14992020400050014 PMC5593096 15035561

[B13] Gokhale S. Lahoti S. Caplan L. R. (2013). The neglected neglect: Auditory neglect. *JAMA Neurol.* 70 1065–1069. 10.1001/jamaneurol.2013.155 23778964

[B14] Gutschalk A. Dykstra A. (2015). Auditory neglect and related disorders. *Handb. Clin. Neurol.* 129 557–571. 10.1016/B978-0-444-62630-1.00031-7 25726290

[B15] Häusler R. Levine R. A. (2000). Auditory dysfunction in stroke. *Acta Otolaryngol.* 120 689–703. 10.1080/000164800750000207 11099144

[B16] Heilman K. M. Valenstein E. Watson R. T. (2000). Neglect and related disorders. *Semin. Neurol.* 20, 463–470. 10.1055/s-2000-13179 11149702

[B17] Jenkinson M. Bannister P. Brady M. Smith S. (2002). Improved optimization for the robust and accurate linear registration and motion correction of brain images. *Neuroimage* 17 825–841. 10.1006/nimg.2002.1132 12377157

[B18] Jenkinson M. Beckmann C. F. Behrens T. E. J. Woolrich M. W. Smith S. M. (2012). FSL. *Neuroimage* 62 782–790. 10.1016/j.neuroimage.2011.09.015 21979382

[B19] Joliot M. Jobard G. Naveau M. Delcroix N. Petit L. Zago L. (2015). AICHA: An atlas of intrinsic connectivity of homotopic areas. *J. Neurosci. Methods* 254 46–59. 10.1016/j.jneumeth.2015.07.013 26213217

[B20] Karnath H. O. Himmelbach M. Rorden C. (2002). The subcortical anatomy of human spatial neglect: Putamen, caudate nucleus and pulvinar. *Brain* 125 350–360. 10.1093/brain/awf032 11844735

[B21] Kriegstein K. Griffiths T. D. Thompson S. K. McAlpine D. (2008). Responses to interaural time delay in human cortex. *J. Neurophysiol.* 100 2712–2718. 10.1152/jn.90210.2008 18799604 PMC2585401

[B22] Lehrl S. (2005). *Mehrfach-wortschatz-intelligenztest: MWT-B.* Frederick, MD: Spitta.

[B23] Lynn G. E. Gilroy J. Taylor P. C. Leiser R. P. (1981). Binaural masking-level differences in neurological disorders. *Arch. Otolaryngol.* 107, 357–362. 10.1001/archotol.1981.00790420031007 7224965

[B24] Nasreddine Z. S. Phillips N. A. Phillips N. A. Bédirian V. Charbonneau S. Whitehead V. (2005). The montreal cognitive assessment, MoCA: A brief screening tool for mild cognitive impairment. *J. Am. Geriatr. Soc.* 53 695–699. 10.1111/j.1532-5415.2005.53221.x 15817019

[B25] National Institute of Neurological Disorders and Stroke [NIHSS] (2019). *NIH stroke scale.* Bethesda, MD: National Institute of Neurological Disorders and Stroke. Available online at: https://www.ninds.nih.gov/stroke-scales-and-related-information

[B26] Neff W. D. Diamond I. T. Casseday J. H. (eds) (1975). *Behavioral studies of auditory discrimination: Central nervous system.* Berlin: Springer International Publishing.

[B27] Pickles J. O. (2013). *An introduction to the physiology of hearing*, 4 Edn. Leiden: Brill.

[B28] Robert Koch-Institut (2017). *12-Month prevalence of stroke or chronic consequences of stroke in Germany. RKI-Bib1.* Berlin: Robert Koch-Institut.

[B29] Rorden C. Brett M. (2000). Stereotaxic display of brain lesions. *Behav. Neurol.* 12 191–200. 10.1155/2000/421719 11568431

[B30] Sang-Bae K. Byung-Woo Y. (2013). Mechanisms of Functional Recovery from Stroke. *Front. Neurol. Neurosci.* 32 1–8. 10.1159/000346405 23859958

[B31] Shinn-Cunningham B. G. Best V. (2008). Selective attention in normal and impaired hearing. *Trends Amplif.* 12 283–299. 10.1177/1084713808325306 18974202 PMC2700845

[B32] Sitek K. R. Gulban O. F. Calabrese E. Johnson G. A. Lage-Castellanos A. Moerel M. (2019). Mapping the human subcortical auditory system using histology, postmortem MRI and in vivo MRI at 7T. *Elife* 8:e48932. 10.7554/eLife.48932 31368891 PMC6707786

[B33] Smith S. M. (2002). Fast robust automated brain extraction. *Hum. Brain. Mapp.* 17 143–155. 10.1002/hbm.10062 12391568 PMC6871816

[B34] Spierer L. Bellmann-Thiran A. Maeder P. Murray M. M. Clarke S. (2009). Hemispheric competence for auditory spatial representation. *Brain* 132 1953–1966. 10.1093/brain/awp127 19477962

[B35] Thiran A. B. Clarke S. (2003). Preserved use of spatial cues for sound segregation in a case of spatial deafness. *Neuropsychologia* 41 1254–1261. 10.1016/S0028-3932(03)00014-9 12753964

[B36] Thompson S. K. Kriegstein K. Deane-Pratt A. Marquardt T. Deichmann R. Griffiths T. D. (2006). Representation of interaural time delay in the human auditory midbrain. *Nat. Neurosci.* 9 1096–1098. 10.1038/nn1755 16921369

[B37] Tissieres I. Crottaz-Herbette S. Clarke S. (2019). Implicit representation of the auditory space: Contribution of the left and right hemispheres. *Brain Struct. Funct.* 224 1569–1582. 10.1007/s00429-019-01853-5 30848352

[B38] Trapeau R. Schönwiesner M. (2015). Adaptation to shifted interaural time differences changes encoding of sound location in human auditory cortex. *Neuroimage* 118 26–38. 10.1016/j.neuroimage.2015.06.006 26054873

[B39] Yarkoni T. Poldrack R. A. Nichols T. E. van Essen D. C. Wager T. D. (2011). Large-scale automated synthesis of human functional neuroimaging data. *Nat. Methods* 8 665–670. 10.1038/nmeth.1635 21706013 PMC3146590

[B40] Znamenskiy P. Zador A. M. (2013). Corticostriatal neurons in auditory cortex drive decisions during auditory discrimination. *Nature* 497 482–485. 23636333 10.1038/nature12077 PMC3670751

