# Corrigendum: Effects of acute ischemic stroke on binaural perception

**DOI:** 10.3389/fnins.2023.1143063

**Published:** 2023-02-01

**Authors:** Anna Dietze, Peter Sörös, Matthias Bröer, Anna Methner, Henri Pöntynen, Benedikt Sundermann, Karsten Witt, Mathias Dietz

**Affiliations:** ^1^Department of Medical Physics and Acoustics, University of Oldenburg, Oldenburg, Germany; ^2^Cluster of Excellence “Hearing4all”, University of Oldenburg, Oldenburg, Germany; ^3^Department of Neurology, School of Medicine and Health Sciences, University of Oldenburg, Oldenburg, Germany; ^4^Research Center Neurosensory Science, University of Oldenburg, Oldenburg, Germany; ^5^Institute of Radiology and Neuroradiology, Evangelisches Krankenhaus Oldenburg, Oldenburg, Germany

**Keywords:** binaural hearing, psychoacoustics, brain lesions, lateralization, binaural masking level difference, magnetic resonance imaging, stroke

In the published article, there was a typographical error. In the study by Bernstein and Trahiotis (2011), stronger lateralization was perceived for the same ILD magnitude and higher-frequency signals, not for lower-frequency signals as stated in the original version of the manuscript. A correction has been made to Section 3.5.1 Control group, Paragraph 1. The corrected paragraph is below.

“Physically left-favoring, to consecutively more right-favoring stimuli, were perceived from the left to the right inside the participants' heads for the ILD and ITD stimuli for all control subjects, with only slight deviations. Apparently, the chosen ILDs, ranging from −12 to 12 dB did not lead to strongly lateralized auditory images (responses close to response keys 1 = left and 9 = right). Previous studies already demonstrated that the extent of perceived lateralization for ILDs of this magnitude varies across subjects (Baumgärtel and Dietz, 2018). It also depends on frequency, with stronger lateralization perceived for the same ILD magnitude and higher-frequency signals (Bernstein and Trahiotis, 2011). Auditory space was distributed roughly symmetrically around zero ITD/ILD, being reflected in the average perceived position over all ILD and ITD stimuli (*mean*) of 5.2 in the control group. Even in the control group, the perceived intracranial positions were not perfectly distributed around the center (5.0). Monaural left or right stimulation was perceived close to the most lateralized intracranial positions (*mon left*: 1.5 and *mon right*: 8.6) with almost no intra-individual variability. For all ILDs and all absolute ITDs ≤ 600 μs, a small variability in single trials can be seen. The standard deviation of given responses was for all stimuli approximately in the range of one response key for the control subjects (e.g., 1.1 for *diotic std*., the standard deviation of zero ILD/ITD). Only one person of the control group produced much more variable data. The variability of ITDs of ±1500 μs was larger than for smaller ITDs in most control subjects. This unnaturally large ITD was perceived less lateralized compared to smaller absolute ITDs. Based only on the center frequency (500 Hz), one cannot distinguish between a time shift of −500 or +1500 μs, as the period at this frequency is 2000 μs. However, since the stimulus is a white noise of 333 Hz bandwidth centered around 500 Hz, the auditory system can partially resolve this ambiguity, by exploiting either the interaural correlation at other frequencies or the envelope ITD. The range of lateralization was larger for ITDs (5.5) compared to ILDs (3.7) and for both interaural differences was much smaller than the maximal possible range of 8”.

The authors apologize for this error and state that this does not change the scientific conclusions of the article in any way. The original article has been updated.
