# Supplementary figures and images for: Effects of acute ischemic stroke on binaural perception
Source: Front Neurosci. 2022 Dec 22;16:1022354. doi: 10.3389/fnins.2022.1022354 (PMC9817147; doi:10.3389/fnins.2022.1022354)

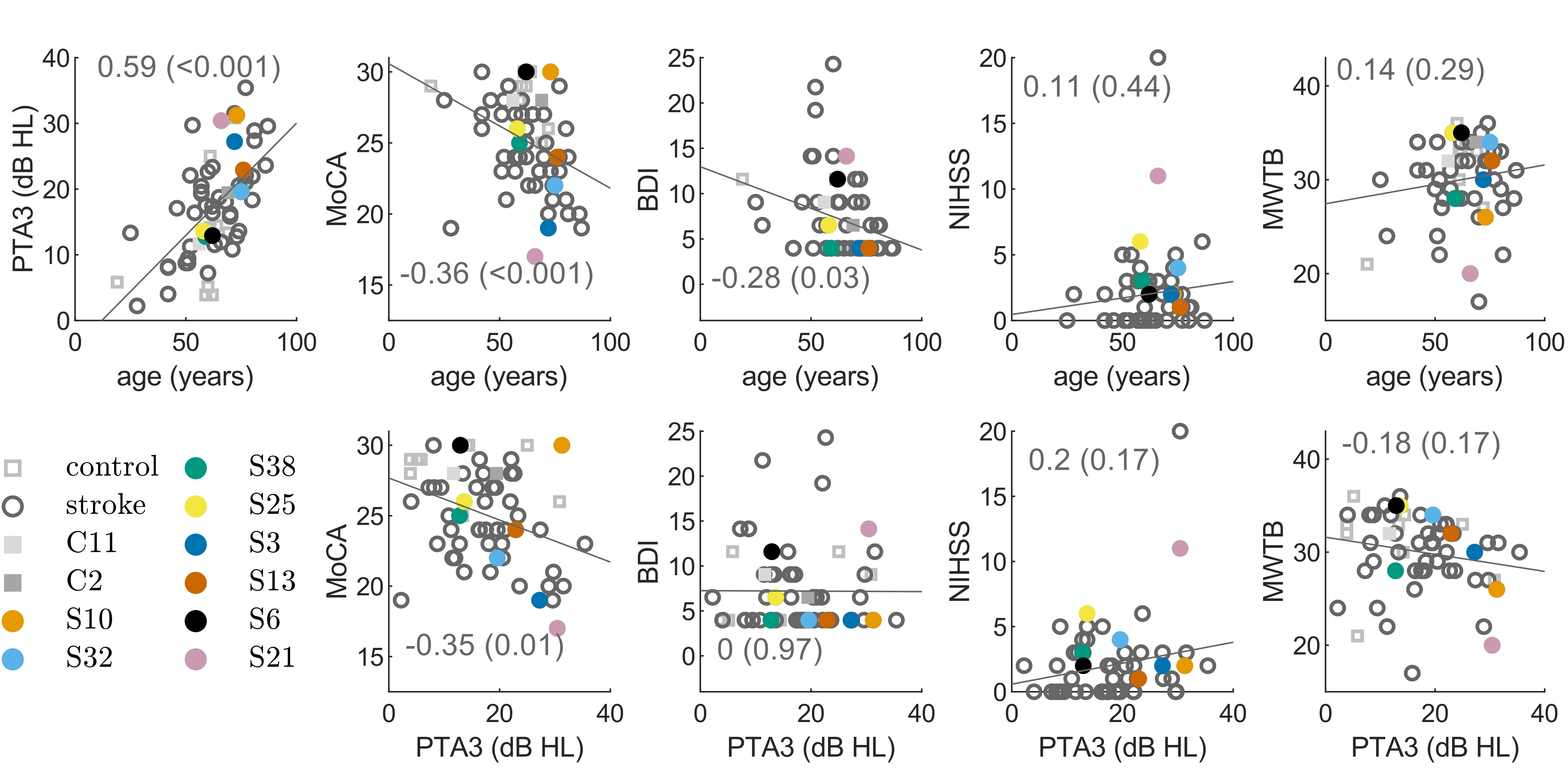

Supplement: Supplementary Figure 1 — Scatter plots representing correlations between age and PTA3 thresholds and the results of the non-auditory measurements (MoCA, NIHSS, BDI, and MWT-B) for the control group (squares) and the stroke group (circles). In each subpanel, linear-regression lines, the Pearson correlation coefficient ρ, and the respective p-value are shown in the form “ρ (p-value)”. Selected participants are highlighted by the color coding used throughout the figures. [file Image_1.JPEG]

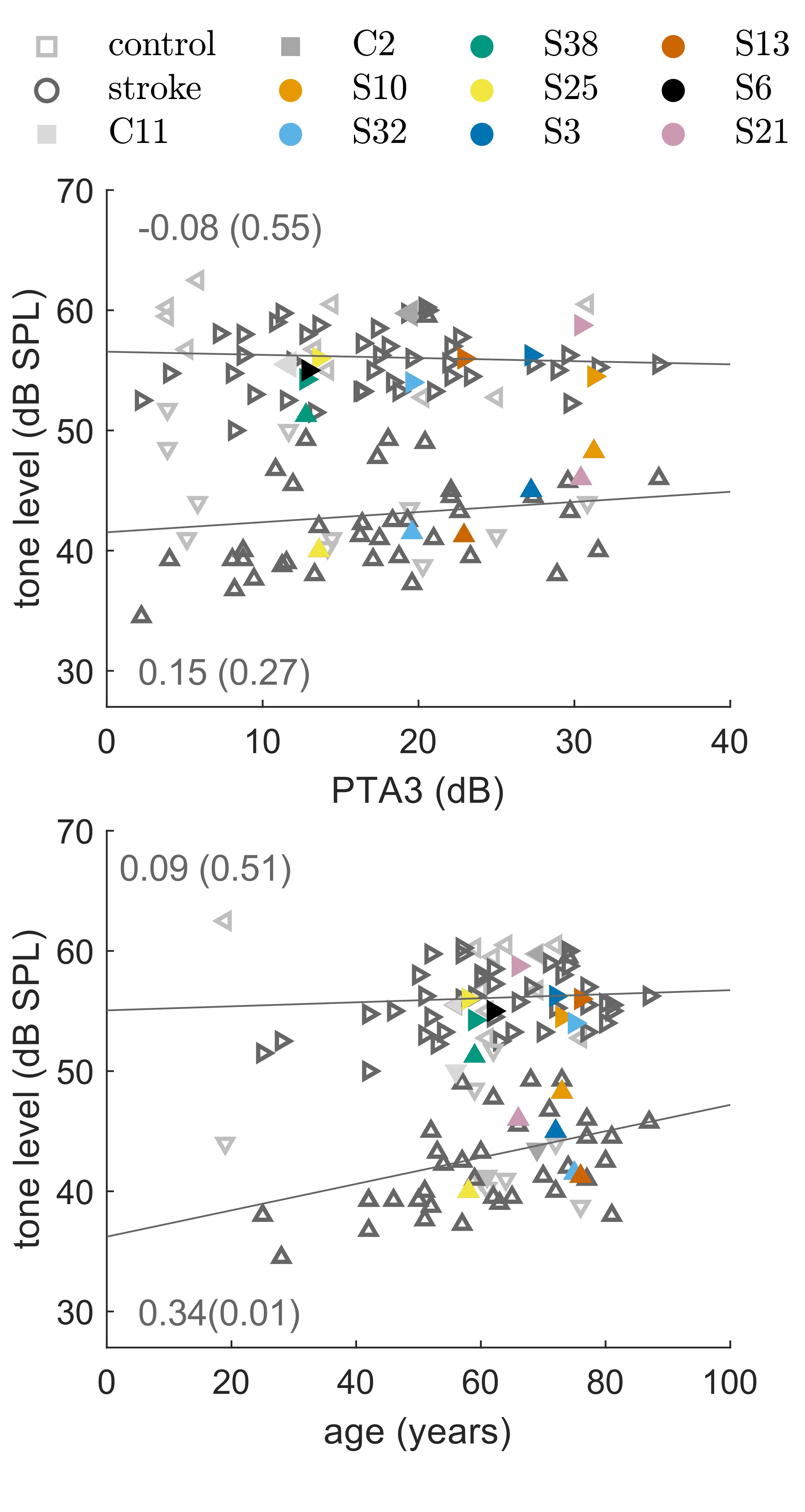

Supplement: Supplementary Figure 2 — Results of the binaural tone-in-noise detection experiment. Tone-in-noise detection thresholds for N0Sπ condition (up- and downward triangles for stroke and control subjects, respectively) and N0S0 condition (left- and right-pointing triangles) over PTA3 (panel A) and over age (panel B). In each subpanel, linear-regression lines, the Pearson correlation coefficient ρ, and the respective p-value are shown in the form “ρ (p-value)”. Selected participants are highlighted by the color coding used throughout the figures. [file Image_2.jpg]

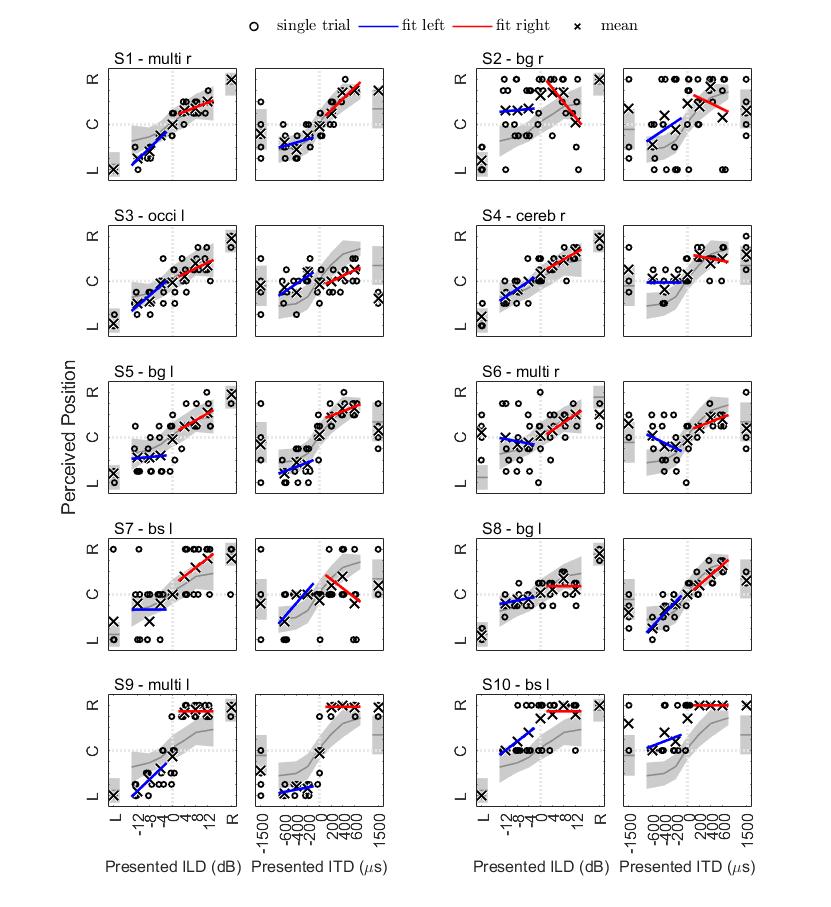

Supplement: Supplementary Figure 3 — Results of the lateralization task for patients S1–S10. The circles represent the responses given to the individual trials of the same stimulus, except for the discarded first trial. The black crosses indicate the means of the given responses. The red and blue lines represent linear fits to right-favoring and left-favoring stimuli, respectively. The gray line and shaded area indicate the mean and the 1.5 times standard deviation interval around the mean response of the control subjects. [file Image_3.JPEG]

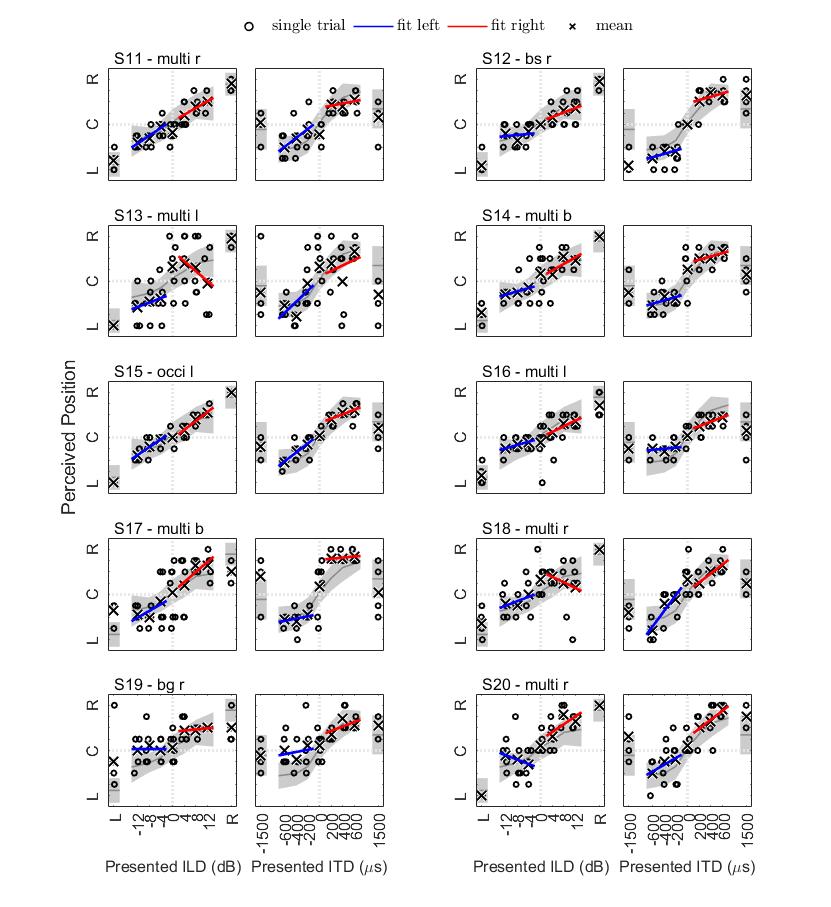

Supplement: Supplementary Figure 4 — Results of the lateralization task for patients S11–S20. The circles represent the responses given to the individual trials of the same stimulus, except for the discarded first trial. The black crosses indicate the means of the given responses. The red and blue lines represent linear fits to right-favoring and left-favoring stimuli, respectively. The gray line and shaded area indicate the mean and the 1.5 times standard deviation interval around the mean response of the control subjects. [file Image_4.JPEG]

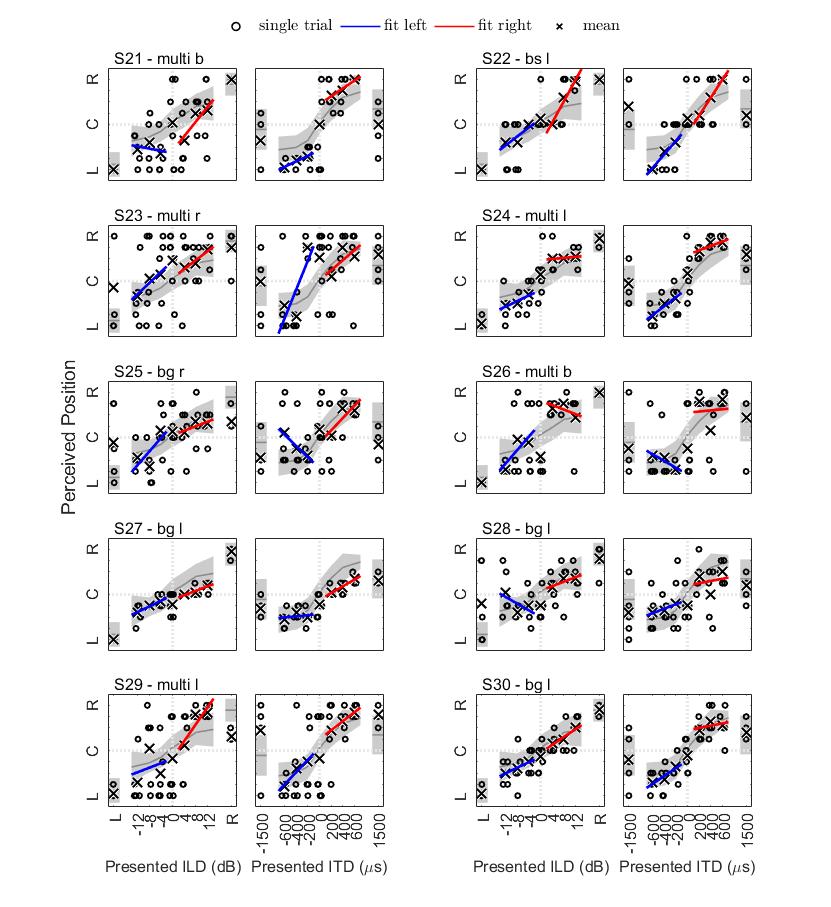

Supplement: Supplementary Figure 5 — Results of the lateralization task for patients S21–S30. The circles represent the responses given to the individual trials of the same stimulus, except for the discarded first trial. The black crosses indicate the means of the given responses. The red and blue lines represent linear fits to right-favoring and left-favoring stimuli, respectively. The gray line and shaded area indicate the mean and the 1.5 times standard deviation interval around the mean response of the control subjects. [file Image_5.JPEG]

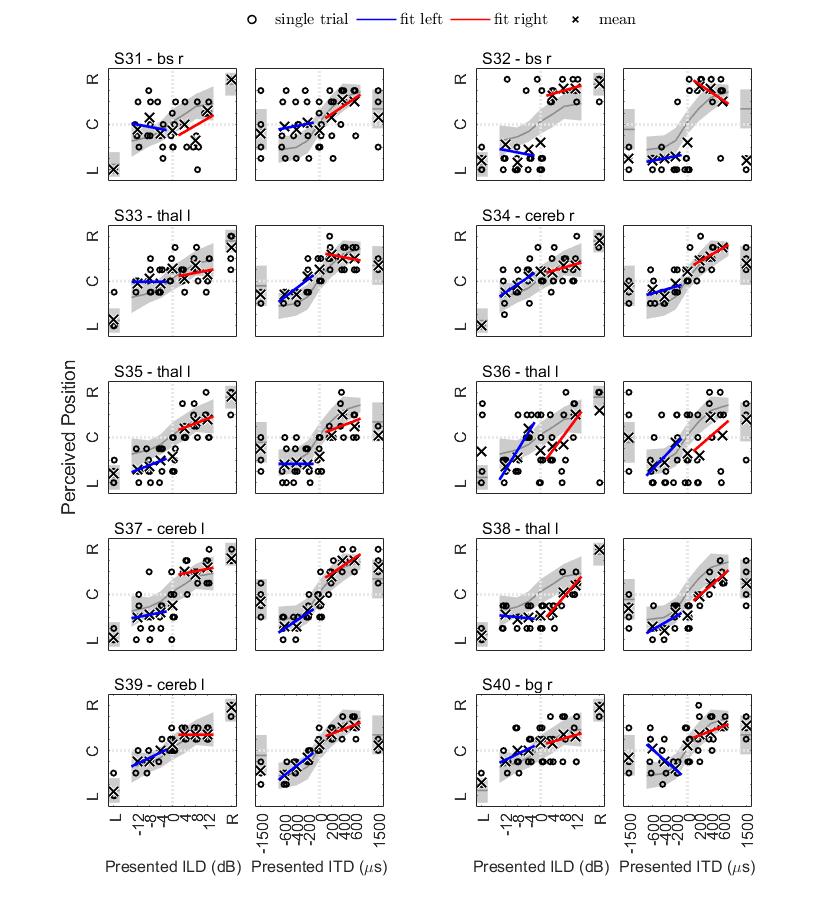

Supplement: Supplementary Figure 6 — Results of the lateralization task for patients S31–S40. The circles represent the responses given to the individual trials of the same stimulus, except for the discarded first trial. The black crosses indicate the means of the given responses. The red and blue lines represent linear fits to right-favoring and left-favoring stimuli, respectively. The gray line and shaded area indicate the mean and the 1.5 times standard deviation interval around the mean response of the control subjects. [file Image_6.JPEG]

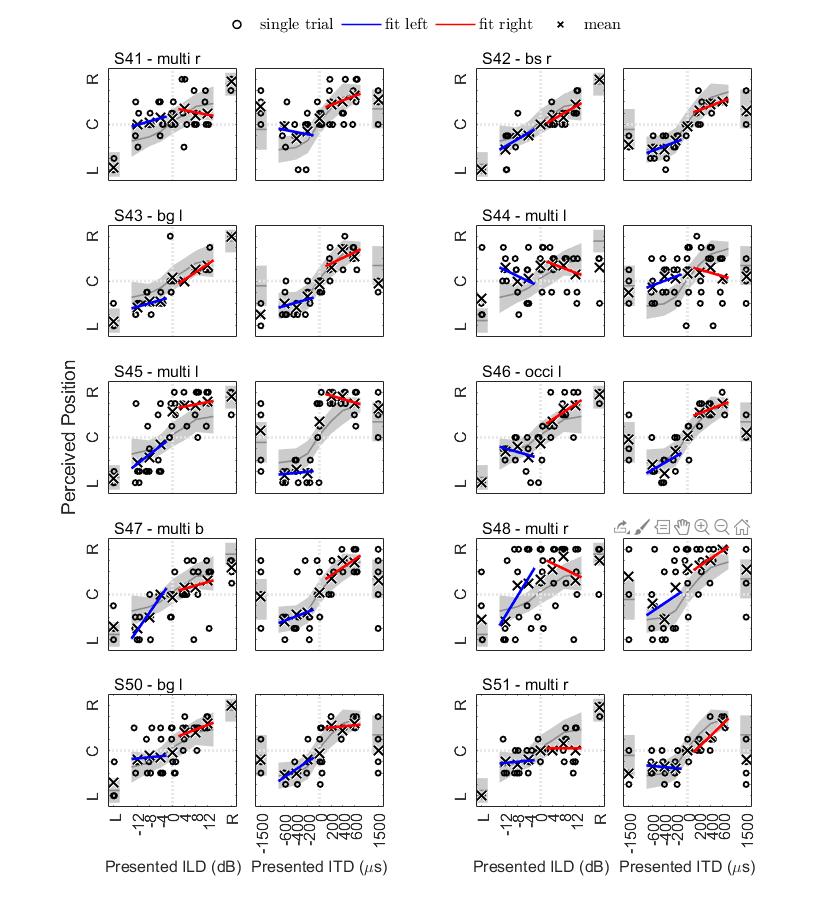

Supplement: Supplementary Figure 7 — Results of the lateralization task for patients S41–S50. The circles represent the responses given to the individual trials of the same stimulus, except for the discarded first trial. The black crosses indicate the means of the given responses. The red and blue lines represent linear fits to right-favoring and left-favoring stimuli, respectively. The gray line and shaded area indicate the mean and the 1.5 times standard deviation interval around the mean response of the control subjects. [file Image_7.JPEG]

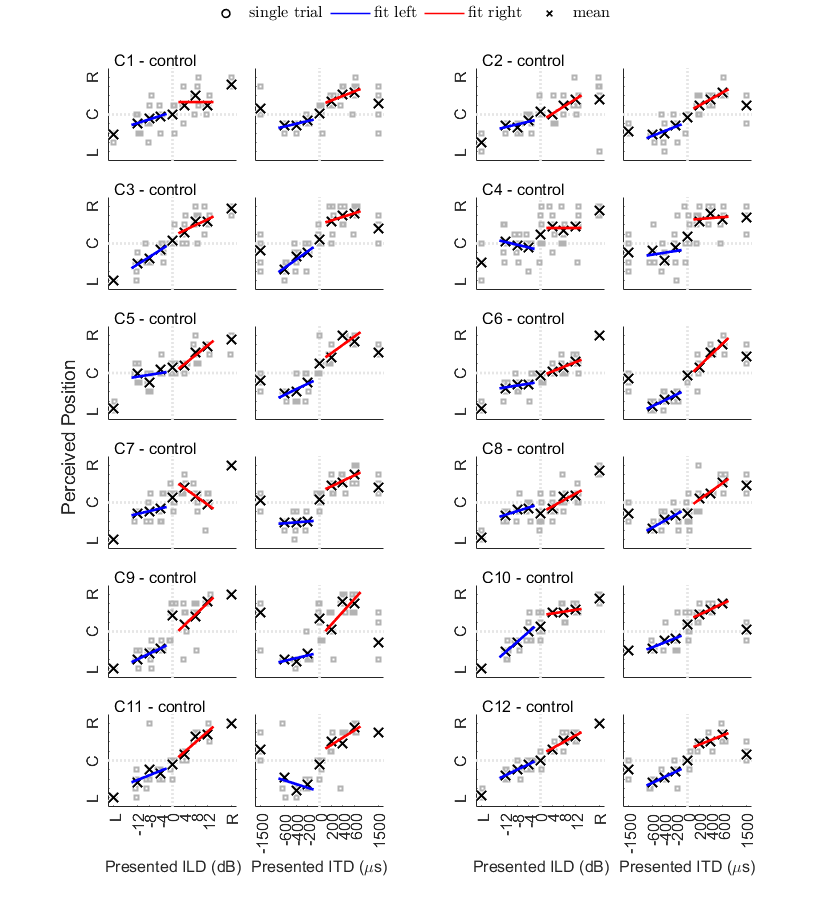

Supplement: Supplementary Figure 8 — Results of the lateralization task for control subjects C1–C12. The squares represent the responses given to the individual trials of the same stimulus, except for the discarded first trial. The black crosses indicate the means of the given responses. The red and blue lines represent linear fits to right-favoring and left-favoring stimuli, respectively. [file Image_8.PNG]
